# Malfunctioning of the Iron–Sulfur Cluster Assembly Machinery in *Saccharomyces cerevisiae* Produces Oxidative Stress via an Iron-Dependent Mechanism, Causing Dysfunction in Respiratory Complexes

**DOI:** 10.1371/journal.pone.0111585

**Published:** 2014-10-30

**Authors:** Mauricio Gomez, Rocío V. Pérez-Gallardo, Luis A. Sánchez, Alma L. Díaz-Pérez, Christian Cortés-Rojo, Victor Meza Carmen, Alfredo Saavedra-Molina, Javier Lara-Romero, Sergio Jiménez-Sandoval, Francisco Rodríguez, José S. Rodríguez-Zavala, Jesús Campos-García

**Affiliations:** 1 Lab. Biotecnología Microbiana, Instituto de Investigaciones Químico-Biológicas, Universidad Michoacana de San Nicolás de Hidalgo, Morelia, Michoacán, México; 2 Lab. de Bioquímica, Instituto de Investigaciones Químico-Biológicas, Universidad Michoacana de San Nicolás de Hidalgo, Morelia, Michoacán, México; 3 Facultad de Ingeniería Química, Universidad Michoacana de San Nicolás de Hidalgo, Morelia, Michoacán, México; 4 Centro de Investigación y de Estudios Avanzados del IPN, Unidad Querétaro, Querétaro, México; 5 Departamento de Bioquímica, Instituto Nacional de Cardiología, México, D.F., México; National Institute of Environmental Health Sciences, United States of America

## Abstract

Biogenesis and recycling of iron–sulfur (Fe–S) clusters play important roles in the iron homeostasis mechanisms involved in mitochondrial function. In *Saccharomyces cerevisiae*, the Fe–S clusters are assembled into apoproteins by the iron–sulfur cluster machinery (ISC). The aim of the present study was to determine the effects of ISC gene deletion and consequent iron release under oxidative stress conditions on mitochondrial functionality in *S. cerevisiae*. Reactive oxygen species (ROS) generation, caused by H_2_O_2_, menadione, or ethanol, was associated with a loss of iron homeostasis and exacerbated by ISC system dysfunction. *ISC* mutants showed increased free Fe^2+^ content, exacerbated by ROS-inducers, causing an increase in ROS, which was decreased by the addition of an iron chelator. Our study suggests that the increment in free Fe^2+^ associated with ROS generation may have originated from mitochondria, probably Fe–S cluster proteins, under both normal and oxidative stress conditions, suggesting that Fe–S cluster anabolism is affected. Raman spectroscopy analysis and immunoblotting indicated that in mitochondria from *SSQ1* and *ISA1* mutants, the content of [Fe–S] centers was decreased, as was formation of Rieske protein-dependent supercomplex III_2_IV_2_, but this was not observed in the iron-deficient *ATX1* and *MRS4* mutants. In addition, the activity of complexes II and IV from the electron transport chain (ETC) was impaired or totally abolished in *SSQ1* and *ISA1* mutants. These results confirm that the ISC system plays important roles in iron homeostasis, ROS stress, and in assembly of supercomplexes III_2_IV_2_ and III_2_IV_1_, thus affecting the functionality of the respiratory chain.

## Introduction

The iron–sulfur centers (Fe–S) are prosthetic groups in many prokaryote and eukaryote enzymes with redox, catalytic, and regulatory functions. These centers are assembled by the iron–sulfur cluster assembly system (ISC), which has been extensively studied and is known to be involved in the incorporation of the Fe–S centers into apoproteins in both bacteria and eukaryotes [1]–[5]. In eukaryotes, two main systems of Fe–S-protein biogenesis have been described, the cytosol/nucleus (CIA) and mitochondrial (ISC) machineries. Functionally, the CIA machinery depends on the mitochondrial ISC machinery [6]–[7]. The ISC assembly machinery for maturation of all cellular Fe–S dependent-proteins (mitochondrial, cytosolic, and nuclear) is also involved in iron homeostasis in prokaryotes and eukaryotes [5], [7]. In *Saccharomyces cerevisiae*, the mitochondrial ISC machinery is encoded by the genes *NFS1*, *ISU1*, *ISU2*, *ISA1*, *ISA2*, *JAC1*, *SSQ1*, *YAH1, GRX5*, and *IBA57* [5], [8]. In yeast, the Ssq1 chaperone and the Jac1 J-protein (co-chaperone) function together to assist in the biogenesis of Fe–S centers of Fe–S-dependent proteins. The Fe–S cluster assembly in mitochondria is initiated by cysteine desulfurase (Nfs-Isd11), which obtains a sulfur group from a cysteine and transfers it to the scaffold proteins Isu1 and its redundant Isu2 protein, assisted by Yah1. This interaction also involves frataxin (Yfh1), which acts as an iron donor or activity regulator. ATPase activity in the Ssq1chaperone is stimulated by the J-type co-chaperone Jac1, during the interaction with the scaffold protein Isu1/Isu2 [5]–[7]. The protein Isu1 is a substrate for both Ssq1 and Jac1, while Jac1 and Isu1 cooperatively stimulate the ATPase activity of Ssq1 [3]. The subsequent cluster transference to recipient apoproteins is assisted by glutaredoxin (Grx5) [5], [9].

Recently, the participation of the proteins Isa1 and Isa2 in the maturation of mitochondrial apoproteins containing 4Fe–4S clusters such as aconitase, homoaconitase, and lipoic acid synthase was described in *S. cerevisiae*; this activity is mediated by physical interaction with the Iba57 assembly-protein [5], [10]–[12]. In contrast, Isa1/Isa2 proteins are dispensable for the generation of mitochondrial [2Fe–2S] and cytosolic [4Fe–4S] proteins, because, although the Isa1 and Isa2 proteins are able to bind iron, they are not used as donors for *de novo* assembly of the [2Fe–2S] cluster on the general Fe–S scaffold proteins, Isu1/Isu2 [5], [12]. Upon depletion of the ISC assembly factor Iba57, which specifically interacts with Isa1 and Isa2, or in the absence of the major mitochondrial [4Fe–4S] protein aconitase, iron is accumulated on the Isa proteins, suggesting that the iron bound to the Isa proteins is required for the *de novo* synthesis of [4Fe–4S] clusters in mitochondria and for their insertion into apoproteins in a reaction mediated by Iba57. Taken together, these findings define Isa1/Isa2 and Iba57 as a specialized, late-acting ISC assembly subsystem that is specifically dedicated to the maturation of mitochondrial [4Fe–4S] proteins [12].

Iron handling by mitochondria during ISC biogenesis must be tightly controlled to avoid a deleterious increase in the concentration of free iron. Ferrous (Fe^2+^) and ferric (Fe^3+^) iron catalyze the formation of the highly reactive hydroxyl radical (OH^•^) in the presence of H_2_O_2_ and O_2_^•−^ species through the Haber-Weiss cycle. In mitochondria, these ROS are physiologically produced as by-products of electron transport chain (ETC) activity. Thus, uncontrolled mitochondrial iron homeostasis causes oxidative damage in DNA, lipids, and proteins via the generation of ROS, which in turn further impairs the function of the ETC and leads to cell death [13].

During the dysfunction of the ISC biogenesis system, accumulation of toxic amounts of iron in the mitochondrial matrix occurs due to upregulation of iron transport systems via the activation of the Aft1 transcription factor [14]. Iron accumulation in yeast requires the activation of vacuolar iron transporters as well as the Mrs3/Mrs4 mitochondrial transporters, which play essential roles in both cell iron homeostasis and heme and Fe–S clusters synthesis by shuttling iron into mitochondria [15]–[17]. The relevance of impaired ISC biogenesis in mitochondrial iron overload and ETC dysfunction is reflected in human diseases like Friedreich's ataxia, sideroblastic anemia, and ISCU myopathy, whose development has been associated with defects in human genes coding for proteins involved in ISC biogenesis, such as Frataxin, Glrx5, and IscU, respectively [18]. In this regard, yeast has been a powerful tool to elucidate key molecular aspects of the pathogenesis of these diseases because several steps of ISC biogenesis and recycling are conserved between yeast and higher eukaryotes [19].

On the other hand, the antioxidant defenses, iron homeostasis and Fe-S recycling are important mechanisms to restore the function and integrity of the cell under oxidizing conditions. We have recently demonstrated that the deletion of genetic components of the mitochondrial ISC assembly machinery in *S. cerevisiae* exacerbates ethanol toxicity via increased ROS generation, provoking depletion of the antioxidant response and leading to apoptosis [20]. Given the central role of mitochondria in iron handling, the role of free iron from Fe–S-containing proteins in ROS production, and the fact that ethanol increases mitochondrial ROS generation, it can be hypothesized that excessive free iron exacerbates the toxicity of ethanol and other stressors in mitochondria by disrupting the functionality of the ETC. To test this hypothesis, we have analyzed the relationship between mitochondrial free iron and the levels of ROS generated by stressors, the amount of Fe–S containing proteins, and their effect on respiratory chain functionality in yeast mutants of the ISC system and affected in mitochondrial iron transport.

## Materials and Methods

### Yeast strains, growth conditions, and survival tests

The haploid *S. cerevisiae* BY4741 (Mat a, *his3*Δ, *leu2*Δ0, *met1*5Δ0, *ura3*Δ0) and its *KanMX4* interruption gene mutants, *ssq1*Δ, *grx5*Δ, *isa1*Δ, *atx1*Δ, *mrs4*Δ, and *aft1*Δ were obtained from Open Biosystems. Growth tests were carried out using yeast extract peptone dextrose (YPD) culture medium. Tubes or flasks were prepared with 10 or 50 mL of YPD culture medium and added stressor (H_2_O_2_, menadione, or ethanol from Sigma), at the indicated concentrations. Culture medium was inoculated with overnight yeast cultures that had reached an optical density of 0.1 at 600 nm (OD_600 nm_) and incubated at 30°C with low-speed shaking (50 rpm). Yeast growth (biomass) was spectrophotometrically monitored at OD_600 nm_. A survival test was carried out in yeast cultures grown on liquid YPD medium, collected in the late exponential growth phase and then adding ethanol 10% (v/v) and 10 mM 1, 10-phenanthroline, incubating at 30°C with low-speed shaking (50 rpm). Cell survival was determined by Trypan blue staining, and yeast counts were performed using a Neubauer chamber [20].

### Real-time quantification of ROS in *S. cerevisiae* cultures

Intracellular ROS in yeast cultures or cell suspensions were determined using oxidant-sensitive, cell-permeant fluorescent probes and fluorescence was quantified by flow cytometry [20]. Cell cultures were grown to the late exponential phase and samples (100 µL) were loaded with the appropriate fluorescent probe. For mitochondrial ROS determination (mit-ROS, mainly H_2_O_2_), yeast suspensions were incubated with 5 µg mL^−1^ of dihydrorhodamine 123 (DHR123; Sigma) and for superoxide (O_2_^•−^) determination, yeast were incubated with 5 µg mL^−1^ dihydroethidium (DHE, Molecular Probes, Invitrogen), at 30°C for 2 h in the dark. Then, yeast cell samples were taken to 1 mL with PBS buffer (NaCl 137 mM, KCl 2.7 mM, Na_2_HPO_4_·2 H_2_O 8.1 mM, KH_2_PO_4_ 1.76 mM, at pH 7.4) and the fluorescence was immediately quantified by flow cytometry using a BD Accuri C6 Flow Cytometer (BD Biosciences). The populations of cells for each of the treatments were gated in the forward scatter and side scatter dot plots to eliminate dead cells and cell debris. Populations corresponding to auto- or basal-fluorescence were located in the left quadrant and cells with emission of fluorescence increments of at least one log unit value were located in the right quadrant of the dot plots. In addition, the percentage of fluorescent cells (PFC) and the median fluorescence intensity (FI) were determined in the monoparametric histograms of fluorescence emission obtained from the dot plots and labeled as percentage of cells and as relative units of fluorescence. The equipment was calibrated using Spherotech 8-peak (FL1-FL3) and 6-peak (FL-4) validation beads (BD Accuri). Fluorescence of the DHR123 probe was monitored in the emission fluorescence channel FL1 (533/30 nm), and for the DHE probe, in the FL2 channel (587/40 nm). A minimum of 20,000 cellular events were analyzed for each determination point. For stressor treatments and Fe^2+^ dose-response assays, yeast cultures grown on YPD medium (10 mL) were loaded with the fluorescent probes by incubating for 30–60 min, washed with PBS and supplemented with the respective concentrations of ROS-generator compounds or Fe^2+^ solution [FeSO_4_(NH_4_) with an equimolar amount of citric acid, Sigma]. At the respective times, samples (100 µL) were harvested, washed and suspended in PBS, adjusting the volume to 1 mL or 1×10^7^ cells mL^−1^ and the fluorescence was determined by flow cytometry.

### Real-time quantification of Fe^2+^

Iron in the yeast suspensions was determined using the fluorescent, cell-permeable indicator for heavy metals Phen green FL (PGFL; Molecular Probes, Invitrogen), which can be used to detect a broad range of ions, including Cu^2+^, Cu^+^, Fe^2+^, Hg^2+^, Pb^2+^, Cd^2+^, Zn^2+^, and Ni^2+^. Fluorescence of PGFL disappears after binding of free Fe^2+^. Therefore, once cells were loaded with the probe, Fe^2+^ was detected by the addition of 1 mM of the chelator 1,10-Phenanthroline. This treatment leads to PGFL-Fe complex dissociation, producing fluorescence [21]. Yeast cells suspensions (1×10^7^ cells/mL) were incubated with PGFL (5 µg/mL) at 30°C for 2 h in darkness. Then, yeast cells were harvested, washed once, and re-suspended in PBS. Fe^2+^ quantification in yeast suspensions was performed without and with ROS-generator treatment and fluorescence was quantified by flow cytometry monitoring the emission fluorescence in channel FL1 (533/30 nm).

### Confocal microscopy of yeast suspensions

*Saccharomyces cerevisiae* YPD-grown cultures were harvested and suspended in PBS at 1×10^7^ cell/mL and loaded with the fluorescent probe DHE or PGFL as detailed above, incubating with light shaking in darkness. Suspensions were treated with and without ethanol (10%) and incubated for 30 min at 30°C. Afterwards, the cell suspensions were incubated with Rhodamine 123 (Rho123; Sigma) during 30 min for mitochondrial co-localization and analyzed using a confocal microscope (Olympus FV1000). The signal evaluating fluorescence emission was observed between 560–580 nm for DHE, between 405–505 nm for PGFL and between 590–600 nm for Rho123. Images were acquired with different magnifications.

### Mitochondria isolation

For the determination of mitochondrial complexes activity, mitochondria of *S. cerevisiae* were isolated from cultures grown in liquid medium YPD at 30°C in a shaking incubator, using a previously described method with light modifications [22], Lyticase from *Arthrobacter luteus* (Sigma-Aldrich) was used instead of zymolyase. Yeast cells were harvested in late exponential growth phase by centrifugation at 2,750× *g* for 15 min at 4°C and washed thrice using distilled water and suspended in digestion solution (sorbitol 1.2 M, EGTA 1 mM, Tris-HCl 50 mM, DTT 10 mM, at pH 7.5); Lyticase was added at 2 mg g^−1^ weight for spheroplast generation. Yeast suspensions were incubated for 60 min at 30°C. Spheroplasts were washed twice with spheroplast washing buffer (sorbitol 1.2 M, EGTA 1 mM, Tris-HCl 50 mM, DTT 10 mM, at pH 7.5). Then, spheroplasts were suspended in homogenizing buffer (sorbitol 0.6 M, HEPES-KOH 20 mM, DTT 10 mM, at pH 7.4) and lysed in a Potter-Elvehjem pestle and glass tube and washed thrice with the same buffer. The unruptured cells were removed by centrifugation at 2,500× *g* for 10 min at 4°C, and yeast mitochondria were harvested from the supernatant by centrifugation at 9,600× *g* for 10 min at 4°C and suspended in homogenizing buffer.

### Determination of *in situ* mitochondrial oxygen consumption rate

Cells (25 mg wet weight) of *S. cerevisiae* were placed in 2.5 mL of MES-TEA buffer (pH 6.0) in a sealed glass chamber with constant stirring. The oxygen consumption rate (OCR) was measured with a Clark-type oxygen electrode coupled to a biological oxygen monitor (YSI 5300). Basal oxygen consumption (state 4), was induced by adding 20 mM glucose as substrate, and 3 min later, 5 µM of the uncoupling agent carbonyl cyanide m-chlorophenyl hydrazone (CCCP) was added to stimulate maximal OCR (uncoupled (U) state). To discriminate the mitochondrial oxygen consumption from unspecific-cytosolic oxygen utilization, the mitochondrial ETC was inhibited with 1 µg antimycin A and a further addition of 0.5 mM KCN [23].

### Determination of the ETC complexes activity

Detergent solubilization of mitochondria for determination of ETC activities was carried out by mixing 250 µL of intact mitochondria (10 mg of protein) plus 750 µL of hypotonic buffer (KCl 100 mM, MgCl_2_ 10 mM, Tris-base 10 mM, pH 7.5, and Triton X-100 (0.02%) with vigorous shaking in a vortex for 15 sec. This solution was centrifuged at 18,600× *g* for 15 min at 4°C. Supernatants were discarded and the pellets suspended in buffer composed of 50 mM KH_2_PO_4_, pH 7.6 and protein was quantified by the Biuret method. These suspensions of permeabilized mitochondria were used to determine the activity of the ETC complexes, as described below.

#### Determination of complex II activity

The activity of complex II was evaluated by measuring the succinate-DCIP oxidoreductase activity of solubilized mitochondria [22]. The reaction mixture contained 0.1 mg/mL permeabilized mitochondria, 1 µg antimycin A and 0.75 mM KCN in a final volume of 1 mL 50 mM KH_2_PO_4_ buffer (pH 7.6). After 5 min of incubation with ETC inhibitors, the determination was started by adding 80 µM 2,6 dichlorophenolindophenol (DCIP) and the basal absorbance at 600 nm was determined for 1 min. Then, the reaction was started by adding 10 mM sodium succinate and the changes in absorbance were further followed for 5 min. The rate of DCIP reduction was calculated from the slopes of the absorbance plots using a molar extinction coefficient for DCIP of 21 mM^−1^ cm^−1^.

#### Determination of complex III activity

For this purpose, the activity of antimycin A-sensitive succinate-cytochrome *c* oxidoreductase was measured, which is representative of complex III activity, using endogenous ubiquinol-6 as substrate [24]. Solubilized mitochondria (0.1 mg/mL) were resuspended in 50 mM KH_2_PO_4_ buffer (pH 7.6) and incubated for 5 min with 0.75 µM KCN. Then, 1.5 mg oxidized cytochrome *c* was added and the basal absorbance was recorded at 550 nm. After 1 min, 10 mM succinate was added, and the reduction of cytochrome *c* was recorded for 3 min. The reaction was stopped by adding antimycin A (1 µg). The rate of cytochrome *c* reduction was determined from the slopes of the absorbance plots, using a molar extinction coefficient for cytochrome *c* of 19.1 mM^−1^ cm^−1^ [22]. Alternatively, complex III activity was measured with 10 mM glycerol, instead of succinate, to bypass electron transfer at complex II and eliminate the possibility that impaired electron transfer at complex II level might mask defects in electron transfer at complex III.

#### Determination of the complex IV activity

Cytochrome *c* oxidase activity was measured in 0.1 mg/mL solubilized mitochondria suspended in 50 mM KH_2_PO_4_ buffer (pH 7.6), incubated for 5 min with 1 µg antimycin A. The reaction was started by adding dithionite-reduced cytochrome *c* (250 µg) and the changes in the absorbance at 550 nm were followed during 1 min. The reaction was stopped with 0.75 mM KCN. The rate of cytochrome *c* oxidation was determined from the slope of the absorbance plots using a molar extinction coefficient for cytochrome *c* of 19.1 mM^−1^ cm^−1^ [22].

#### Determination of lactate-cytochrome c oxidoreductase

This activity was measured in 1 mg/mL freeze-thawed mitochondria re-suspended in 50 mM KH_2_PO_4_ buffer incubating for 5 min with 0.75 µM KCN. Basal absorbance at 550 nm during 1 min was registered, and then the reaction was started by adding 10 mM D-lactic acid. Then, the changes in the absorbance were recorded for 5 min. The rate of cytochrome *c* reduction was determined from the slopes of the absorbance plots using a molar extinction coefficient for cytochrome *c* of 19.1 mM^−1^ cm^−1^.

### Determination of *cis*-aconitase activity

Aconitase activity was determined as described by Henson and Cleland (1967) [25]; 100 µg of mitochondrial protein was suspended in lysis buffer (Tris-HCl 50 mM, Triton X-100 0.02%, pH 7.4) with vigorous shaking and incubated for 5 min. Extracts were centrifuged at 9,900× *g* for 5 min at 4°C, and supernatants obtained were used for *cis*-aconitase determinations. Eighty micrograms of protein from the supernatants were used for enzymatic determination in reaction buffer (Tris-HCl 90 mM, isocitrate 20 mM, pH 7.4) with light agitation; the absorbance at 240 nm was immediately recorded.

### Mitochondrial membrane potential

Membrane potential in the mitochondrial suspensions was determined using the fluorescent, cell-permeable indicator Rho123. Mitochondrial suspensions (1×10^7^ mitochondria/mL) were loaded with Rho123 (5 µg/mL) and incubated at 30°C for 30 min in darkness. Suspensions were harvested, washed once and re-suspended in PBS. Membrane potential in suspensions was determined by fluorescence generation and quantified by flow cytometry, monitoring the emission fluorescence in channel FL1 (533/30 nm).

### Raman spectroscopy of mitochondria

Suspensions of intact mitochondria (250 µg) from yeast cultures grown on YPD were subjected to Raman spectroscopy. Raman analysis was performed using a microRaman spectrometer (Dilor model LabRam) equipped with a confocal microscope with 50× amplification, using He-Ne laser emitting at 632.8 nm and 30 mW at sample point for excitation. Mitochondrial dried-pellets were collocated in a cooper plate and laser impacted into a spot of 2 µm with an integration time of 60 s; a 256×1024 pixel charge-coupled device (CCD) was used as a photon detector. The spectra showed correspond to the average of spectra overlapped by 60 s of recording; measurements were carried out at room temperature with sample preparation as described elsewhere [26].

### Native Gel Electrophoresis and western blot

For Blue Native gel electrophoresis, samples of 100 µg of mitochondrial protein were solubilized using buffer A containing dodecylmaltoside (1 g/g), triton X-100 (2.4 g/g), and digitonin (3 g/g) as described [27], and separated by native polyacrylamide gel electrophoresis on 8% Bis-tris gels (BN-PAGE); mitochondrial complexes were identified as described [28]–[30]. For immunodetection assay 50 µg of mitochondrial protein were run in SDS-PAGE 12% polyacrylamide gels and transferred to polyvinylidene difluoride (PVDF) membranes. Membranes were blocked using dry milk in PBS-T and blotted with the *S. cerevisiae* anti-Rip1 antibody as first antibody in blocking medium at a 1∶20000 dilution for 2 h at 4°C [4]; after washing, the membrane was incubated with the secondary antibody, a monoclonal anti-mouse IgG HRP-conjugate (Promega), in blocking medium at a 1∶5000 dilution for 2 h at 4°C; the membrane was washed with PBS-T and developed using Supersignal West Pico Luminol (Pierce) and exposing in light-sensitive films. Assays were conducted by triplicate and representative images are shown. Bands intensities in gels or films were quantified using the Image J software.

## Results

### ROS susceptibility of *S. cerevisiae* is exacerbated by mutations in the *ISC*

**Figure 1:**
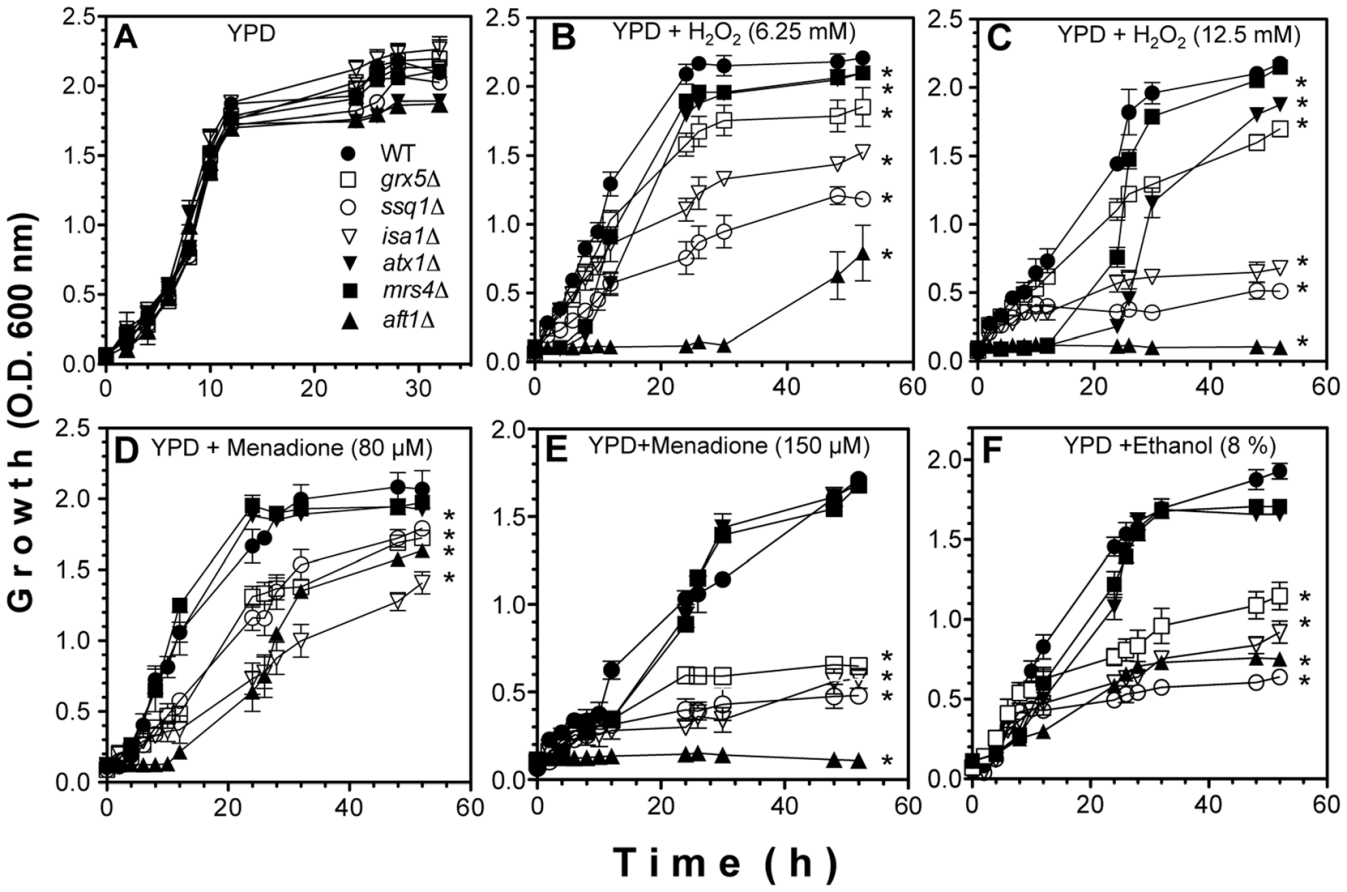
Growth kinetics of *S. cerevisiae ISC* mutants in the presence of ROS generators. A–E) Yeast cultures were grown on YPD liquid medium as follows: A) no addition, B) H_2_O_2_ 6.25 mM, C) H_2_O_2_ 12.5 mM, D) menadione 80 µM, E) menadione 150 µM, and F) ethanol 8% (v/v). Cultures were incubated at 30°C with light shaking and growth (biomass) was determined by measuring O.D. at 600 nm. Values representing the mean and standard errors of the mean (SEM) are indicated as bars (n = 3), one-way ANOVA with Bonferroni's post-hoc test was used to compare mutants versus controls. Significant differences (*p*<0.05) are indicated with (*).

In order to verify whether the dysfunction in the ISC system is related to a parallel increase in the sensitivity to oxidative damage, the susceptibility to several ROS-generating compounds was tested using three *ISC* mutants, whose disrupted genes encode proteins that play important roles in assembly of Fe–S centers. In addition, control *S. cerevisiae* strains that display a severe imbalance in iron homeostasis were used; these included *atx1*Δ mutants, which are impaired in high affinity iron-depleted medium, as this gene is involved in copper trafficking and delivery to Fet3p, which oxidizes Fe^2+^ to Fe^3+^ for uptake by Ftr1p [31] and *mrs4*Δ mutants, which show cellular iron accumulation and sensitivity to H_2_O_2_ and menadione, as this gene is co-regulated with the iron regulon, and encodes the Mrs4 Fe^2+^ iron transporter at the inner mitochondrial membrane under conditions of iron deprivation [15]–[17]. *aft1*Δ mutant shows increased ROS sensitivity and iron accumulation by inducing iron-sensing genes under iron depletion conditions [32]. All these yeast strains show iron-dependence when grown in the presence of phenanthroline, which induces iron-depletion, and which was improved with iron addition in the culture media (Figure S1). *aft1*Δ mutant is more sensitive to iron depletion than *mrs4*Δ mutant, which in turn correlates with the exacerbated sensitivity of the *aft1*Δ mutant to ROS inducers (Fig. 1). Of the *ISC* mutants, *ssq1*Δ, *grx5*Δ, and *isa1*Δ mutants showed a significantly impaired growth rate compared to WT, displaying severely compromised growth at concentrations in the range of 6.25–12.5 mM H_2_O_2_. *grx5*Δ mutants were the least sensitive at all concentration of H_2_O_2_ (Fig. 1b–c). The susceptibility to menadione (a superoxide generator), followed a similar pattern to that observed with H_2_O_2_ treatments: at 80 µM menadione, *ISC* mutants showed a moderate but significant inhibition in their growth kinetics, with respect to the WT strain (Fig. 1d), while at 150 µM menadione, also with ethanol (8%), the growth of all *ISC* mutants was drastically affected (Fig. 1e–f); again, *grx5*Δ was the least sensitive *ISC* mutant to the stressor. As expected, *atx1*Δ and *mrs4*Δ mutants (which show imbalanced iron homeostasis), showed similar behavior to the WT under iron sufficiency, except in YPD supplemented with 12.5 mM H_2_O_2_ (Fig. 1c), in which delayed growth was observed. In contrast, the hypersensitive *aft1*Δ mutant exhibited a marked sensitivity to H_2_O_2_, menadione, and ethanol treatments (Fig. 1b–f).

### Increment in ROS generation correlates with a dysfunctional ISC assembly system

**Figure 2:**
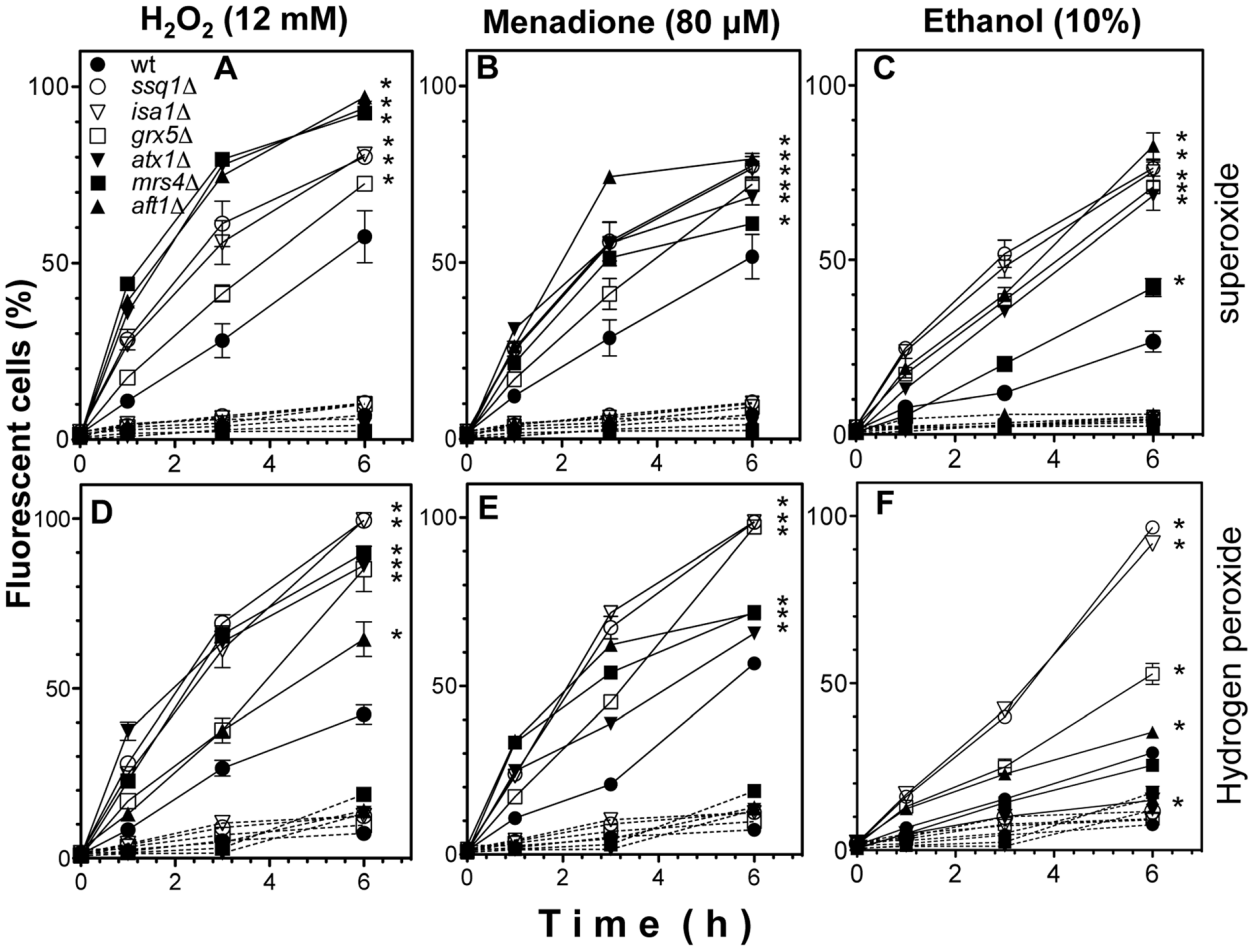
Kinetics of ROS generation in suspensions of *S. cerevisiae ISC* mutants treated with stressors. Yeast cultures were grown in liquid YPD medium without stressors and harvested in late exponential growth phase. Yeast YPD-grown cultures were incubated for 2 h with the respective ROS probe; then, the suspensions were treated with and without stressor (H_2_O_2_ 12 mM, menadione 80 µM, and ethanol 10%), incubated at 30°C with light shaking. Samples (100 µL) were taken and suspended in PBS buffer for determination of intracellular ROS levels by real-time analysis in a flow cytometer. A–F) Results represent the percentage of cells that showed positive fluorescence. Yeast suspensions without a stressor (dashed lines) and with a stressor treatment (continuous lines). The ROS fluorescent probes DHE (O_2_^•−^ indicators) and DHR123 (mitochondrial ROS in general, mainly a H_2_O_2_ indicator) were used. A–C) Fluorescence determination using DHE probe, D–F) fluorescence determination using DHR123 probe. Values are the mean of three independent experiments with 20,000 cells counted by flow cytometry per each point. SEM values are indicated as bars (n = 3), one-way ANOVA was used to compare mutants versus to WT. Significant differences (*p*<0.05) are indicated with (*).

To elucidate whether the enhanced sensitivity to ROS generators was correlated with increased mitochondrial ROS generation due to *ISC* mutations, real-time quantification of ROS was performed by flow cytometry, using the fluorescent ROS indicators DHE and DHRH123 to detect mitochondrial O_2_^•−^ and H_2_O_2_, respectively. ROS generation was determined as the percentage of fluorescent cells (PFC), corresponding to cells that produced ROS level increments of at least one log unit. All mutants displayed a significant increment in the PFC generating O_2_^•−^ or H_2_O_2_ when ROS induction was conducted using H_2_O_2_, menadione, or ethanol (Fig. 2, continuous lines), compared to untreated strains (Fig. 2, dotted lines). PFC values in yeast suspensions without ROS-inducers were approximately 10%, while in treatments with ROS-inducers the PFC increased to 40–90%. Moreover, *ssq1*Δ, *isa1*Δ, and *grx5*Δ mutants showed higher PFC values than the WT in all ROS-inducer treatments (Fig. 2), indicating that *ISC* mutations caused increased ROS generation compared to WT yeast. Importantly, ethanol treatment exacerbated H_2_O_2_ generation in *ISC* mutants, but decreased H_2_O_2_ generation in *atx1*Δ, *mrs4*Δ, and *aft1*Δ iron- transport mutants, compared to *ISC* mutants (Fig. 2f). In addition, *atx1*Δ, *mrs4*Δ, and *aft1*Δ mutants exhibited higher levels of superoxide than H_2_O_2_ (Fig. 2c and 2f). *grx5*Δ mutants, concordant with their lowest sensitivity to oxidants among *ISC* mutants (Fig. 1), also produced the lowest amounts of ROS, except O_2_^•−^, under treatment with 10% ethanol (Fig. 2). In addition, O_2_^•−^ production in *aft1*Δ mutants was higher than in all other *ISC* mutants, concordant with its higher sensitivity to stressors (Fig. 1). Interestingly, when ethanol was used as the ROS generator, the *ISC* mutants produced higher levels of H_2_O_2_ than the iron-transport mutants, (Fig. 2f). These results indicated that the O_2_^•−^ and H_2_O_2_ generation are increased by treatment with ROS inducers in *S. cerevisiae* and are enhanced by the *ISC* mutation in a time-dependent manner, as in the defective iron-transport mutants *atx1*Δ, *mrs4*Δ, and *aft1*Δ.

### Increment in ROS generation correlates with Fe^2+^ release and is increased by *ISC* mutations

**Figure 3:**
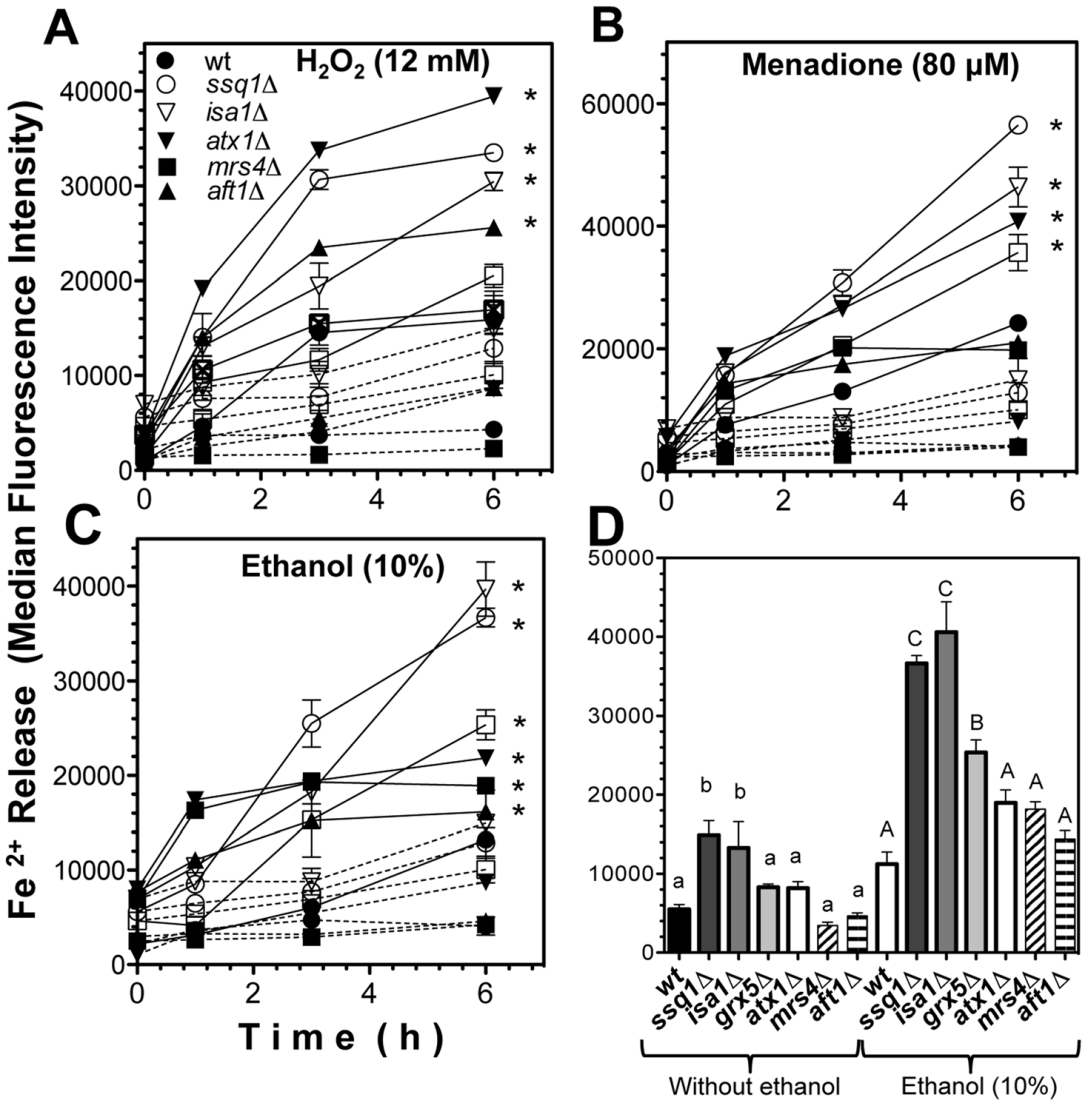
Determination of Fe^2+^ release in *S. cerevisiae ISC* mutants. Yeast cultures were grown in liquid YPD medium, harvested and suspended in YPD at 1×10^7^ cells/mL and charged with the fluorescent probe PGFL and incubated for 2 h at 30°C with light shaking in darkness. Then, yeast suspensions were treated with and without a stressor and incubated at 30°C with light shaking. Samples (100 µL) were collected, suspended in PBS buffer, and the fluorescence intensity in the cells was evaluated by real-time flow cytometry within 6 h. Free Fe^2+^ determination in yeast suspensions without a stressor (dashed lines) and with stressor treatment (continuous lines). A) H_2_O_2_ 12 mM, B) menadione 80 µM, C) ethanol 10% v/v, D) Free Fe^2+^ determination at 6 h of treatment with ethanol (10%). Results represent the fluorescence intensity of yeast cells. Values are the mean of three independent experiments with 20,000 cells counted by flow cytometry per each point. SEM values are indicated as bars (n = 3), one-way ANOVA with Bonferroni's post-hoc test was used to compare mutants to controls. Significant differences (*p*<0.05) are indicated with (*) for (A–C). Tukey's post-hoc test was used for (D), and significant differences (*p*<0.05) with respect to the WT control are indicated with different letters for the treatments; lowercase and uppercase letters indicate without and with ethanol treatment, respectively.

To evaluate whether the increment in ROS following treatment with oxidant agents was correlated with the functionality of ISC assembly system and the level of the free iron pool, *in vivo* real-time free Fe^2+^ quantification was performed using flow cytometry. Cell suspensions of *ssq1*Δ, *grx5*Δ, and *isa1*Δ mutants showed a significant, time-dependent increment in levels of free Fe^2+^ compared to the WT strain, independent of the presence or absence of oxidants (Fig. 3). As expected, yeast suspensions treated with toxic concentrations of H_2_O_2_ (12.5 mM), menadione (80 µM), or ethanol (10%) exhibited higher iron- and time-dependent fluorescence increments than untreated yeast strains, leading to a 2–5 fold augmentation of the quantity of free Fe^2+^ in *ISC* mutants after 6 h of treatment. Notably, *ssq1*Δ and *isa1*Δ mutants showed higher levels of Fe^2+^ than *grx5*Δ mutants. In addition, Fe^2+^ release was higher in *atx1*Δ mutants than in *aft1*Δ and *mrs4*Δ mutants, and in these mutants, free Fe^2+^ was also increased when they were treated with oxidant agents (Fig. 3). However, *aft1*Δ and *mrs4*Δ mutants showed behavior intermediate between the WT strain and *ISC* mutants; these cells showed higher free Fe^2+^ than WT, but lower free Fe^2+^ than *ISC* and *atx1*Δ mutants (Fig. 3b–c). When the Fe^2+^ release data were analyzed following 6 h of treatment with ethanol, a significant increment in fluorescence values was observed for *ssq1*Δ, *grx5*Δ, and *isa1*Δ strains, but not for the iron-transport defective strains *atx1*Δ, *mrs4*Δ, and *aft1*Δ. However, in the absence of ethanol, only *ssq1*Δ and *isa1*Δ mutants showed significant differences in free Fe^2+^ release values (Fig. 3d). As mentioned above, with ethanol treatment, the free Fe^2+^ value was significantly increased in all strains, compared to untreated yeast cultures.

**Figure 4:**
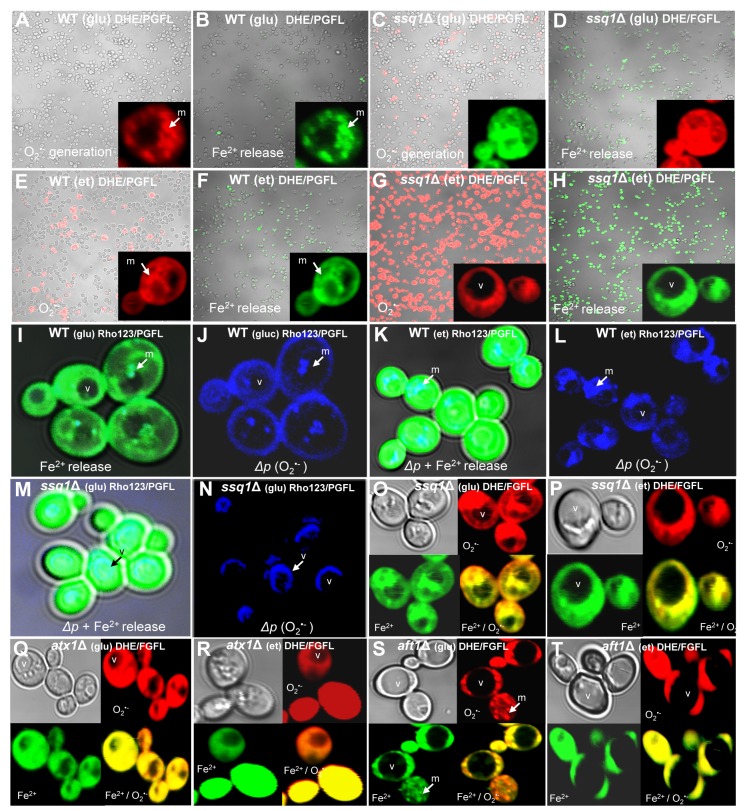
Confocal microscopy images of S. cerevisiae cultures treated with oxidant agents to detect localization of ROS and free Fe^2+^ in intracellular compartments. Yeast YPD-grown cultures were loaded with the fluorescent probe DHE or PGFL and treated with and without ethanol (10%), incubated for 30 min at 30°C and co-loaded with Rho123 as a mitochondrial co-localization marker, and observed using a confocal microscope (Olympus FV1000). (A–D) WT and *ssq1*Δ mutant grown on glucose (2%) and with ethanol (10%) (E–H), using DHE probe for superoxide determination or PGFL probe for free Fe^2+^ determination, as indicated. Cells are shown in boxes, and mitochondria and vacuoles are indicated with (m) and (v). (I–L) Images of WT yeast growth in glucose or treated with ethanol 10% and stained with PGFL and Rhodamine 123 probes, as indicated. (M–P) Images of *ssq1*Δ mutants treated with glucose or ethanol (10%), using PGFL and Rhodamine 123 probes, as indicated. (Q–T) Images of O_2_^•−^ and free Fe^2+^ co-localization in *atx1*Δ and *aft1*Δ mutants grown on glucose or treated with ethanol (10%), and staining with PGFL or DHE probes, as indicated. Superoxide generation areas are shown as fluorescent granules within the cells (see inset of A, J–L), free Fe^2+^ is shown as green cells and green granules within the cells (see inset of B and I), merged images are shown as yellow cells and granules within cells (O–T), and mitochondrial structures are shown as cyan granules within the cells, using the Rho123 probe (I–L). Images of the yeast cells were taken using 10× magnification, 40× magnification, and 65× magnification of yeast cells.

In addition, microscopic analysis showed that ethanol treatment caused an increment in superoxide generation, associated with release of free Fe^2+^ and being exacerbated in *ISC* mutants (Fig. 4). For example, in both WT cells and *ssq1*Δ mutants, O_2_^•−^ levels were higher under treatment with ethanol than with glucose; this response was exacerbated in *ssq1*Δ mutants, consistent with iron-fluorescence levels determined using the PGFL probe, indicating that it was associated with free Fe^2+^ release (Fig. 4a–h). Interestingly, in the WT strain grown on glucose, O_2_^•−^ production and free Fe^2+^ were co-localized in mitochondrial structures (Fig. 4a–b), which were defined by high fluorescence in the *ssq1*Δ mutants or in yeast treated with ethanol (Fig. 4c–h). Further co-localization assays were performed, using rhodamine 123 as an indicator of the mitochondrial membrane potential (Δ*p*). Images showed that in the WT strain, free Fe^2+^ fluorescence was observed in all cells, but with greatest intensity in mitochondrial structures, co-localized with O_2_^•−^ generation; interestingly, high-intensity fluorescence was observed inside the cytoplasmic membrane, a response that intensified under ethanol treatment (Fig. 4i–l). As expected, in *ssq1*Δ mutants, which showed affected respiration and Δ*p* behavior (see below), high free Fe^2+^ fluorescence was observed, but it was not co-localized with mitochondrial activity; however, it was probably associated with vacuolar structures (Fig. 4m–n). These results suggest that at least in the WT, the O_2_^•−^ generation and free Fe^2+^ release occurred in the mitochondria, although other cellular compartments may also have been involved in this effect. Co-localization assays were performed to analyze O_2_^•−^/Fe^2+^ levels in *ISC* and iron-transport mutants. In both *ssq1*Δ, *atx1*Δ and *aft1*Δ mutants, increased levels of fluorescence corresponding to O_2_^•−^ generation and free Fe^2+^ release were observed; in yeast strains, these increased levels were co-localized in possible mitochondrial structures, around some hyper-structures that may correspond to vacuoles (Fig. 4o–t). These results confirm that *ISC* mutations cause an increase in free iron, which was enhanced by treatment with ROS inducers such as ethanol, and which was preferentially associated with mitochondrial structures; interestingly, in *ISC* and iron-transport mutants as well as in WT under ethanol treatment, a clear swelling of vacuolar structures was observed.

**Figure 5:**
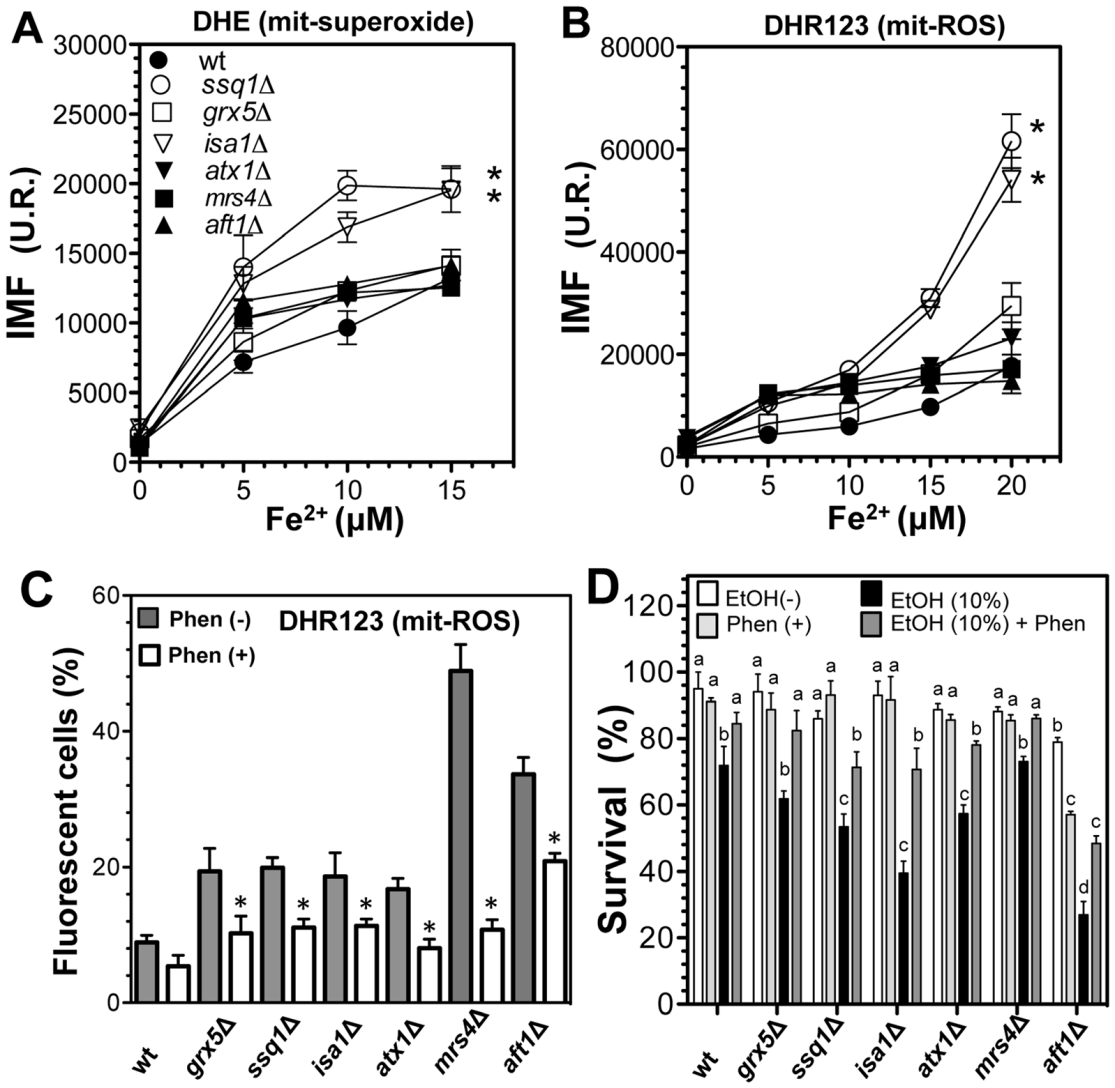
Effect of Fe^2+^ treatment on *S. cerevisiae ISC* mutants. YPD-grown yeast cultures were harvested and suspended in YPD at 1×10^7^ cell/mL, loaded with ROS-sensitive probes, and subjected to Fe^2+^ dose-response assays. Fluorescence intensity was determined by flow cytometry using DHE probe (A), and DHR123 (B). Yeast suspensions were treated with Fe^2+^ [FeSO_4_(NH_4_)] (10 µM) with and without the metal chelator 1,10-phenanthroline (1 mM), and the percentage of fluorescent cells was determined by flow cytometry (C). Values are the mean of three independent experiments with 20,000 cells counted by flow cytometry for each point. (D) Yeast survival using ethanol as a ROS-inducer. Yeast suspensions were treated with and without ethanol (10%) and with 1,10-phenanthroline (20 mM), and the percentage of surviving cells was determined using Trypan Blue staining; yeast counting was performed using a Neubauer chamber [20]. SEM values are indicated as bars (n = 3), one-way ANOVA with Bonferroni's post-hoc test was used to compare mutants with the control WT strain (A–C), significant differences (*p*<0.05) are indicated by (*); or Tukey's post-hoc for (D), significant differences (*p*<0.05) with respect to the WT control are indicated with different lowercase letters.

To confirm that enhanced ROS generation was correlated with the free Fe^2+^ content, real-time quantification of ROS by flow cytometry in a medium containing sufficient iron was performed using Fe^2+^dose-response tests (Fig. 5). As expected, all yeast strains displayed a significant, dose-dependent increment in levels of fluorescence (indicating O_2_^•−^ and H_2_O_2_ generation) when treated with increased concentrations of Fe^2+^. *ssq1*Δ and *isa1*Δ mutants showed the highest ROS generation; in contrast, *grx5*Δ mutants showed a moderate increment in ROS compared to the WT strain, but this increment was lower than that observed in *ssq1*Δ and *isa1*Δ mutants. For *atx1*Δ, *mrs4*Δ, and *aft1*Δ iron-homeostasis deficient mutants, both the DHE and DHR123 probes showed that the ROS content was similar to those observed in the WT and *grx5*Δ strains (Fig. 5a–b). These results indicated that at Fe^2+^concentrations below 10 µM, O_2_^•−^ was the main species produced, while at concentrations of Fe^2+^ between10–20 µM, an increment of H_2_O_2_ was also observed (Fig. 5a–b). In addition, determination of ROS generation in YPD-grown cultures showed that the ROS increment was significantly decreased in all mutants by addition of the metal chelator phenanthroline, although this effect was not statistically significant in the WT cells (Fig. 5c). These results confirm the notion that free iron is responsible for an important proportion of the ROS generated in both *ISC* and defective iron-transport mutants.

The role of free iron in ROS generation in the presence of ethanol in *ISC* mutants was further confirmed by determination of cell survival in cultures treated with a toxic concentration of ethanol (10%) and the addition of an iron chelator (10 µM phenanthroline). The *ISC* mutants showed decreased survival following ethanol treatment, and *ssq1*Δ and *isa1*Δ mutants were most sensitive; when a Fe^2+^ chelator was added to the cultures, survival was significantly restored in all strains (Fig. 5d). These results are in concordance with an increased free iron content and exacerbation of ROS generation observed in *ISC* mutants. Likewise, phenanthroline also exerted a significant protective effect against toxic ethanol concentrations in the iron homeostasis defective mutants *atx1*Δ, *mrs4*Δ, and *aft1*Δ. These results indicate that the toxic effects produced by ethanol and other oxidant agents were associated with intracellular Fe^2+^ release in a dose-dependent manner, and that this phenomenon is exacerbated by dysfunction of the ISC system.

### Cellular free iron release is correlated with a decrement in Fe-S proteins content

**Figure 6:**
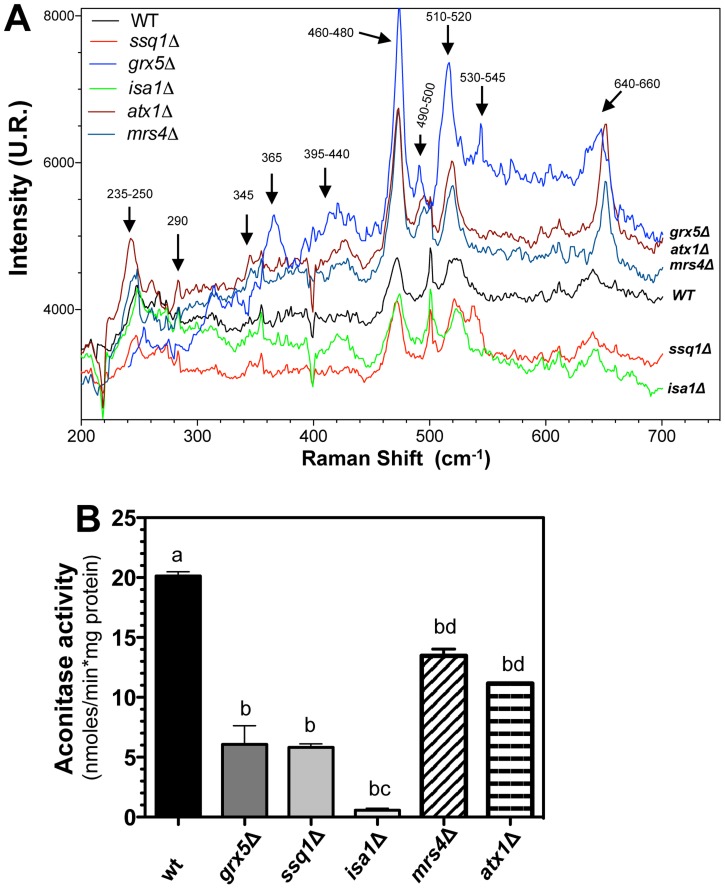
Analysis of mitochondrial Fe–S-containing proteins in *S. cerevisiae ISC* mutants. Cell extracts of yeast cultures grown in YPD to the late exponential growth phase were used to isolate mitochondria, as described in the Materials and Methods section. A) Raman scattering spectra of the mitochondria isolated from *S. cerevisiae ISC* mutants. Raman spectra were recorded at a laser excitation of 632.8 nm with 30 mW. Each spectrum is the average of scans recorded over 60 sec, using photon counting at 0.5 cm^−1^ increment spectral resolution. Bands corresponding to the [2Fe–2S] and [4Fe–4S] clusters are indicated with arrows [26]. B) Enzymatic activity of *cis*-aconitase was determined in mitochondrial suspensions as described in the Materials and Methods. Values are the mean of three independent experiments. SE values are indicated as bars (n = 3), one-way ANOVA with Tukey's post-hoc test was used to compare yeast strains, and significant differences (*p*<0.05) are indicated with different lowercase letters.

The above results led us to hypothesize that the increment in free iron levels induced by oxidative stress and ethanol and exacerbated by ISC system dysfunction arise partially from iron sources such as proteins containing Fe–S centers, including complexes II and III of the ETC. Raman spectroscopy analysis has been used as an analytical tool for determination of Fe–S species and contents; therefore, this technique was utilized to determine the Fe–S content in mitochondria isolated from *ISC* mutants or the iron-transport defective mutants *atx1*Δ and *mrs4*Δ grown in YPD. Signal intensities in the interval 200–700 cm^−1^ at 632.8 nm in the Raman spectra are in agreement with signals corresponding to photonic emission, characteristics of previously described [2Fe–2S] and [4Fe–4S] centers [26], [33]. In our system, mitochondria from *S. cerevisiae* clearly showed Raman signals in stretching regions of peaks at 345–365, 390–440, 460–480, 490–500, 510–520, and 640–660 cm^−1^ (Fig. 6a). The intensity of Raman signals of the Fe–S centers were clearly diminished in mitochondria from *ssq1*Δ and *isa1*Δ mutants compared to WT mitochondria, whereas in *grx5*Δ mutants, the signal peaks showed increased intensities. Interestingly, the iron deficient *atx1*Δ and *mrs4*Δ mutants showed peaks intensities higher than those of the WT. These results indicate that the amount of mitochondrial Fe–S center signals were diminished in mitochondria from *ssq1*Δ and *isa1*Δ mutants, but were overproduced in *grx5*Δ, *atx1*Δ, and *mrs4*Δ mutants.

To investigate the role of Fe-S proteins in Fe^2+^ release under oxidative stress, we conducted a functional analysis of the 4Fe–4S protein *cis*-aconitase, which has been described as ROS-sensitive and as an iron donor for the Fenton reaction. With respect to [2Fe–2S] clusters, we assayed the activity of ETC complex III which is rich in that center and is known to be a main source of O_2_^•−^ generation in mitochondria [34]. *Cis*-aconitase activity was almost totally abolished in the *isa1*Δ mutants compared to the WT strain (Fig. 6b), confirming that in the ISC assembly system, the Isa1 protein is essential for assembly of the [4Fe–4S] cluster into aconitase enzyme. Moreover, aconitase activity was partially inhibited in the remaining *ISC* mutants, whereas in *mrs4*Δ and *atx1*Δ mutants, a decrease in aconitase activity was also observed but to lesser extent that in *ISC* mutants.

However, respiratory complex assembly studies have indicated that the [2Fe–2S]-Rieske protein of complex III is essential for the correct formation of ETC supercomplexes, constituted of complex III (ubiquinol-cytochrome *c* reductase or *bc_1_* complex) and complex IV (cytochrome *c* oxidase) [4], [35].Therefore, we analyzed the assembly of ETC supercomplexes using BN-PAGE gels [27]–[30].

**Figure 7:**
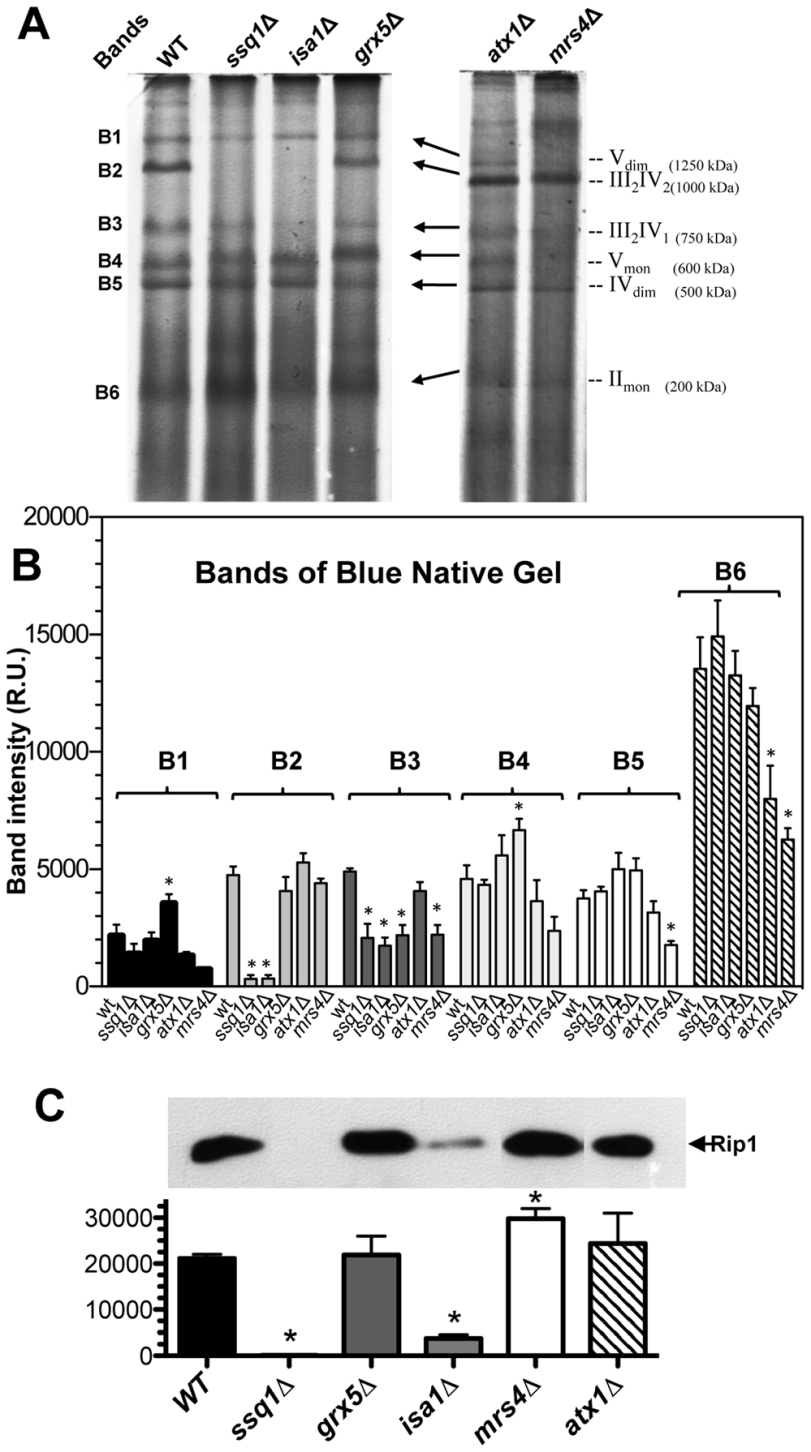
Analyses of supercomplex formation in the mitochondrial ETC of *S. cerevisiae ISC* mutants. To analyze ETC supercomplex formation, mitochondrial suspensions isolated from yeast grown on glucose at the late exponential growth phase were solubilized and the proteins separated using blue native polyacrylamide gel electrophoresis (BN-PAGE) as described in the Materials and Methods [27]–[29]. The ETC mitochondrial supercomplexes and their molecular mass in kilodaltons are indicated to the right of the gel (A) as described elsewhere [27], [28], [30]. B) Analysis of band intensities of the supercomplexes observed in (A). C) Immunoblotting of mitochondrial extracts using anti-Rip1 antibody as the first antibody [4] and monoclonal anti-mouse IgG HRP conjugate as the second antibody; the densitometry analysis plot is shown below. Data correspond to three independent assays determining the band intensity by densitometry analysis using Image J software. Values are the mean of three independent mitochondrial isolations. SE values are indicated as bars (n = 3), one-way ANOVA with Bonferroni's post-hoc test was used to compare yeast strains, and significant differences (p<0.05) with respect to the WT control are indicated by (*).

Interestingly, the results of the BN-PAGE gels indicated that assembly of the III_2_IV_2_ and III_2_IV_1_ supercomplexes is dependent on the functionality of the ISC system. The band corresponding to the III_2_IV_2_ supercomplex was almost absent in mitochondria from *ssq1*Δ and *isa1*Δ mutants, but the band corresponding to the III_2_IV_1_ supercomplex was detected at low levels in all *ISC* mutants (Fig. 7a). Remarkably, in the densitometric analysis of the gels, the intensity of the bands corresponding to the III_2_IV_2_ supercomplex was significantly affected in *ssq1*Δ and *isa1*Δ mutants; the III_2_IV_1_ supercomplex was also diminished in *ssq1*Δ, *isa1*Δ, *grx5*Δ, and *mrs4*Δ mutants, whereas in *atx1*Δ mutant, a response similar to the WT was observed (Fig. 7b). In addition, densitometry data indicated that the content of dimeric complex V (i.e. the F_1_F_0_ ATPase) and its monomer were significantly increased in *grx5*Δ mutants, whereas the dimer of complex IV and II remained unaffected in *ISC* mutants but not in the iron-transporter deficient strains. Immunoblotting assays using anti-Rip1 antibody (Rieske protein) confirmed the supercomplexes formation and Raman spectrometry findings. In western blot analysis of mitochondrial protein extracts from the *S. cerevisiae* strains, the protein signal corresponding to Rip1p was absent from mitochondria from *ssq1*Δ mutant, and significantly diminished in the *isa1*Δ mutant, but in *grx5*Δ, *mrs4*Δ, and *atx1*Δ mutants similar levels to the WT was observed, while *mrs4*Δ mutant showed a stronger signal (Fig. 7c).

### Mitochondrial ETC functionality is affected by *ISC* mutations

**Figure 8:**
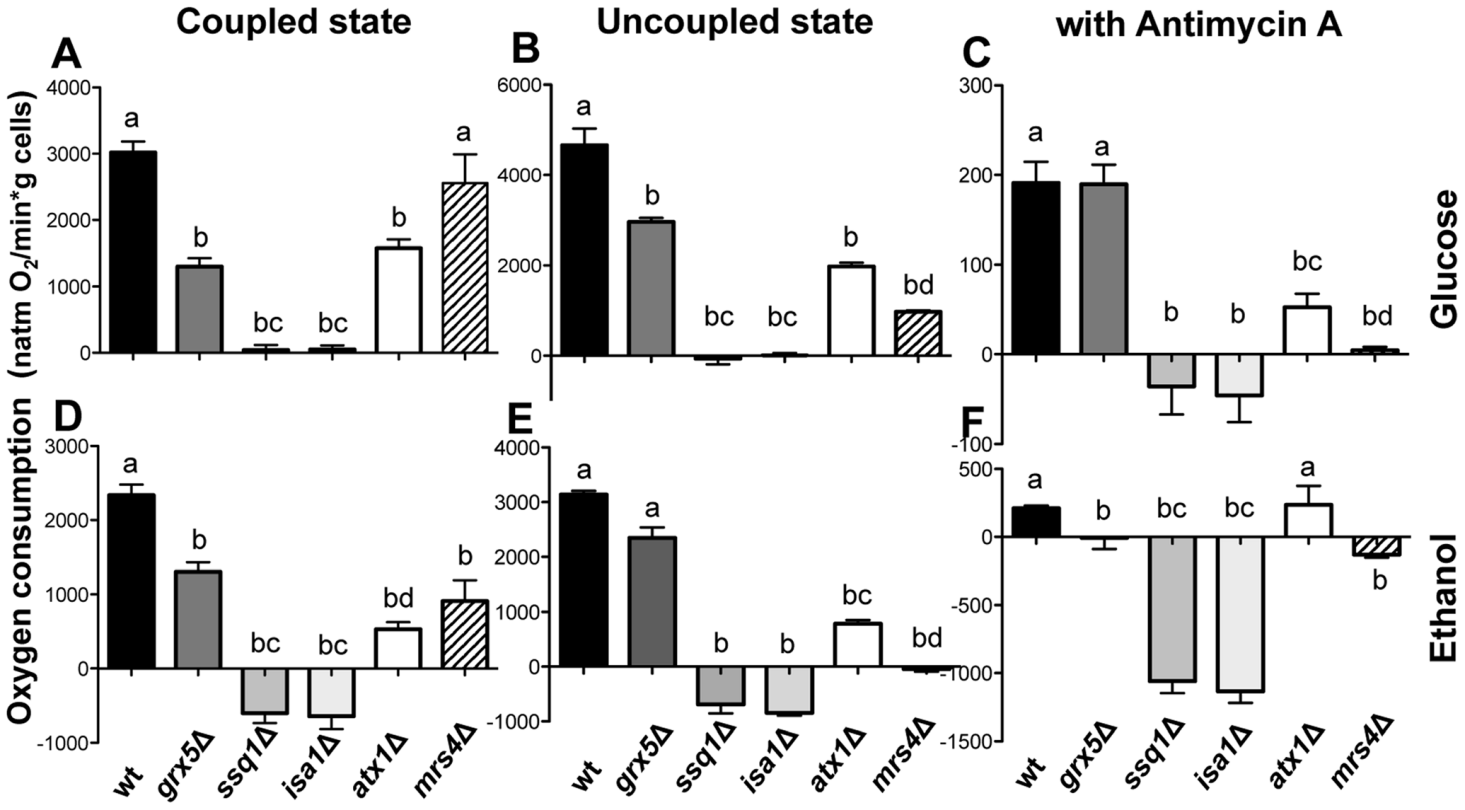
Respiration test of cell suspensions from *S. cerevisiae ISC* mutants. Mitochondrial functionality was evaluated in yeast suspensions obtained from cultures grown in liquid YPD medium, cells were harvested in the late exponential growth phase and re-suspended in MES-TEA buffer with glucose or with ethanol and incubated at 30°C with light shaking. Cells were used for oxygen consumption rate (OCR) measurements with a Clark-type oxygen electrode coupled to a biological oxygen monitor as described in the Materials and Methods. A–C) Basal OCR with glucose as substrate, D–F) with ethanol treatment. A and D) OCR under coupled state conditions, B and E) OCR under uncoupled state conditions using CCCP for uncoupling, C and F) OCR under complex III blocking conditions using antimycin A as an inhibitor. Values are the mean of three independent experiments. SE values are indicated as bars (n = 3), one-way ANOVA with Tukey's post-hoc test was used to compare yeast strains, significant differences (*p*<0.05) are indicated with different lowercase letters.

**Figure 9:**
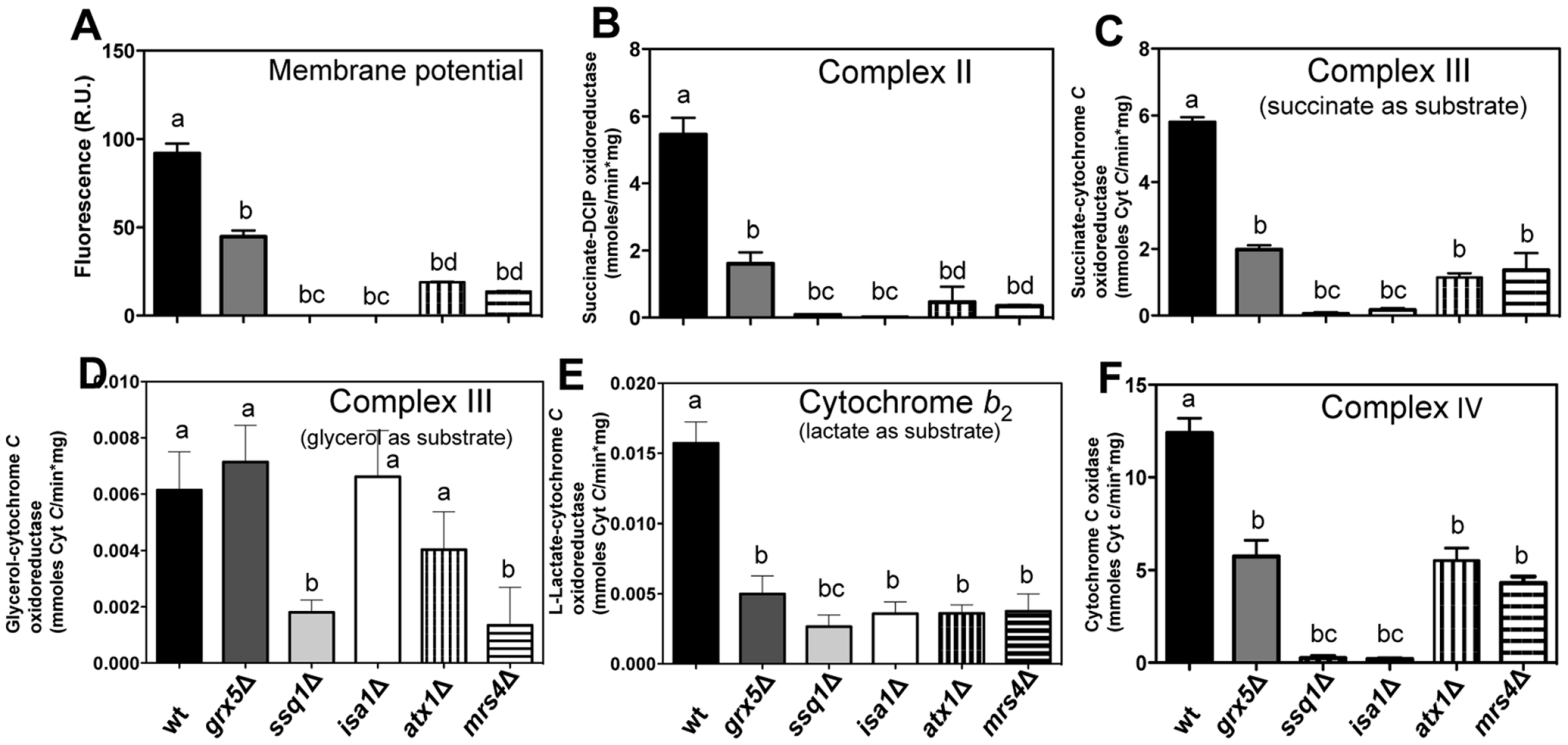
Analyses of the functionality of mitochondrial respiratory chain complexes in *S. cerevisiae ISC* mutants. Mitochondrial functionality was evaluated in mitochondrial suspensions obtained from cultures grown in liquid YPD medium, cells were harvested in the late exponential growth phase, mitochondria were isolated and re-suspended in the appropriate buffer, and mitochondrial activities were measured as described in the Materials and Methods. A) membrane potential, B) activity of succinate-DCIP oxidoreductase, C) activity of succinate-cytochrome *c* oxidoreductase, D) activity of glycerol-cytochrome *c* oxidoreductase, E) activity of L-lactate-cytochrome *c* oxidoreductase, and F) activity of cytochrome *c* oxidase. Values are the mean of three independent experiments. SE values are indicated as bars (n = 3), one-way ANOVA with Tukey's post-hoc test was used to compare yeast strains, and significant differences (*p*<0.05) are indicated with different lowercase letters.

Raman spectroscopy observations of mitochondria isolated from *S. cerevisiae* under iron sufficiency indicated that the [Fe–S] cluster content was diminished in *ssq1*Δ and *isa1*Δ mutants, and native gels showed a clear alteration in the amount of the III_2_IV_2_ and III_2_IV_1_ supercomplexes. We therefore evaluated the *in situ* mitochondrial oxygen consumption rates (OCR) to determinate the functionality of the ETC in *ISC* mutants. In this case, *atx1*Δ and *mrs4*Δ mutants were included as controls, because *aft1*Δ cells showed decreased mitochondrial content and severely impaired function following isolation (data not shown). Respiration was completely abolished in both the coupled and uncoupled states in *ssq1*Δ and *isa1*Δ mutants, whereas in *grx5*Δ, *atx1*Δ, and *mrs4*Δ mutants, the OCR was partially decreased in comparison with the WT strain (Fig. 8a–b). Remarkably, ethanol treatment caused oxygen release in the assay chamber (i.e. negative values for OCR, Fig. 8d–f) instead of oxygen consumption in *ISC* mutants, except for *grx5*Δ and *atx1*Δ. This is suggestive of ROS production, since superoxide dismutase catalyzes the conversion of O_2_^•−^ to O_2_ and H_2_O_2_, while catalase converts the latter species into H_2_O and O_2_. In *S. cerevisiae* mitochondria, complex III is the only site of ROS generation in the ETC, since it lacks a rotenone-sensitive complex I, the other site of ROS production in the ETC of superior eukaryotes [35]. We exposed the cells to antimycin A, an inhibitor of complex III, to further explore the possible role of complex III in ROS generation. In the absence of ethanol, a small amount of O_2_ generation was detected in all *ISC* mutants except in *grx5*Δ, whereas in the presence of ethanol, higher rates of O_2_ generation were detected in these mutants, and O_2_ consumption was fully inhibited in *grx5*Δ cells (Fig. 8f). Conversely, antimycin A-insensitive oxygen consumption was observed in WT, *grx5*Δ, and *atx1*Δ cells in the absence of ethanol, and only in WT and *atx1*Δ cells in the presence of ethanol. In contrast to the behavior of *ISC* mutants, *mrs4*Δ mutant, which shows affected iron homeostasis, displayed an OCR similar to that of the WT strain in the presence of glucose in the coupled state (Fig. 8a). To further corroborate the degree of mitochondrial dysfunction in the various strains, we evaluated mitochondrial membrane potential (Δ*p*). In concordance with their inability to respire, *ssq1*Δ and *isa1*Δ mutants did not exhibit Δ*p* (Fig. 9a). Furthermore, OCR correlated with the magnitude of Δ*p* in WT, *grx5*Δ, *atx1*Δ, and *mrs4*Δ cells, since membrane potential was 2–4-fold higher in WT than in *grx5*Δ or *atx1*Δ and *mrs4*Δ mutants. The OCR in a coupled state followed a similar pattern (Fig. 8a and 9a), which in turn may be related to the decreased complex II signal observed for these mutants (Fig. 7a and 7b).

To determine which segment of the ETC was responsible for the effects described above, partial ETC reactions were analyzed. Succinate-DCIP oxidoreductase activity (representative of complex II activity) was observed to be abolished in *ssq1*Δ and *isa1*Δ mutants, and to be severely affected but not abolished in control mutants *atx1*Δ and *mrs4*Δ, which displayed behavior similar to *grx5*Δ mutants, that showed 30–40% of the WT activity (Fig. 9b).

The same trend was observed in both antimycin A-sensitive succinate-cytochrome *c* oxidoreductase (representative of the activity of complex III, using the endogenous ubiquinol-6 pool as a substrate) [24] and cytochrome *c* oxidase (representative of complex IV) activities; their activities in complexes III and IV were almost entirely abolished in *ssq1*Δ and *isa1*Δ mutants, whereas in *grx5*Δ, *atx1*Δ, and *mrs4*Δ strains, a remnant activity of ∼20–40% with respect to WT mitochondria was detected (Fig. 9c and 9f).

Antimycin A-sensitive succinate-cytochrome *c* oxidoreductase activity is dependent on electron transfer between complex II and complex III. Thus, to eliminate the possibility that impaired succinate-cytochrome *c* oxidoreductase activity was the result of impaired complex II activity, and not of direct damage of complex III, the activity of complex III was tested by reducing the mitochondrial quinone pool with glycerol via the concerted action of porin-associated glycerol kinase and mitochondrial glycerol-3-phosphate dehydrogenase [36], [37]. No differences in this activity with respect to mitochondria from WT cells were observed in *grx5*Δ, *isa1*Δ, or *atx1*Δmutant, while mitochondria from *ssq1*Δ and *mrs4*Δ mutants exhibited a 3-fold diminution (Fig. 9d). Another respiratory enzyme that utilizes cytochrome *c* as its electron acceptor is L-lactate-cytochrome *c* oxidoreductase or cytochrome *b*_2_ [38]. This activity was similar in all the mutants tested; however, it was three-fold lower in the mutants than in the WT strain (Fig. 9e).

## Discussion

The toxic effects of ethanol on mitochondrial function have been attributed to a variety of factors, ranging from alteration of ETC complex activities [39], loss of heme groups from cytochromes [40], oxidative degradation and depletion of mtDNA [41], diminished number of active ribosomes [34], decreased glutathione pools and lipid peroxidation [42]. Although many of these events are related to increased ROS generation, none of these reports have addressed the possibility that disturbances in mitochondrial free iron from Fe–S clusters may be related to ethanol toxicity. Nevertheless, earlier reports have identified a link between ROS overproduction and iron overload in the toxic effects of ethanol, since iron chelation attenuates some of the disturbances in the antioxidant defenses caused by ethanol consumption [43], [44]. We have recently observed in yeast cells that ROS generation during ethanol stress was exacerbated by mutations in *ISC*, which participate in the various steps in Fe–S biogenesis [20]. In the present study, we hypothesized that the increased free iron content was correlated with the dysfunctional mitochondrial Fe–S assembly system, which affects iron homeostasis after treatment with ethanol or ROS generators. The results obtained in the present study indicate that a dysfunctional ISC assembly system increases susceptibility to ethanol and ROS generators, such as H_2_O_2_ and menadione (Fig. 1), probably via both increased mitochondrial ROS levels and disruption of ETC functionality. These effects seem to be linked to an increment in the free iron pool, since iron chelation had a protective effect in ISC mutants exposed to ROS inducers. This suggests a close relationship between levels of free iron and ROS generation, where the Fe–S assembly system plays a relevant role in iron homeostasis and its dysfunction may partially contribute to excessive iron release to the cell.

The sensitivity of the mutants to ROS generators and ethanol, as well as their levels of ROS production and free iron release (Fig. 1–3), appear to be highly dependent on the functionality of the ETC (Figs. 8–9), since strains that showed better respiratory rates, higher activity of ETC complexes and higher mitochondrial transmembrane potentials (i.e. WT and *grx5*Δ cells), also exhibited lower sensitivity to ethanol and ROS generators, presented lower levels of free Fe^2+^, and showed decreased levels of ROS production than mutants with null membrane potential and fully impaired ETCs (i.e. *ssq1*Δ and *isa1*Δ). These results also indicate that a dysfunctional ISC assembly system produces an increased sensitivity to ROS generators. Concordantly, ethanol and other ROS inducers also produce a ROS imbalance in *ISC* mutants in a concentration-dependent manner [20].

The findings obtained using the fluorescent ROS probes DHE and DHR123 (Fig. 2) suggest that ROS are mainly generated and probably accumulated in mitochondria after treatment with toxic concentrations of ROS inducers; an additive effect on ROS generation was observed in *ISC* mutants. In addition, the results obtained for *atx1*Δ, *mrs4*Δ, and *aft1*Δ mutants confirmed that altered iron homeostasis caused an increased generation of ROS (such as superoxide and hydrogen peroxide), which was exacerbated when ethanol, H_2_O_2_, or menadione were used as inducers, suggesting the participation of free iron in the ROS sensitivity (Fig. 2).

Biogenesis of Fe–S centers in *S. cerevisiae* occurs mainly in mitochondria, and the assembly mechanism of these centers depends on the functionality of *ISC* gene products. Iron is an essential component of this process, and its cellular content is dependent on transport systems, chelating proteins and storage. It is well known that iron can be released from Fe–S proteins by O_2_^•−^ or H_2_O_2_ [1], [5], [12], [29], which in turn can lead to the generation of the strongly oxidant OH^•^ radical via Fenton's chemistry.

**Figure 10:**
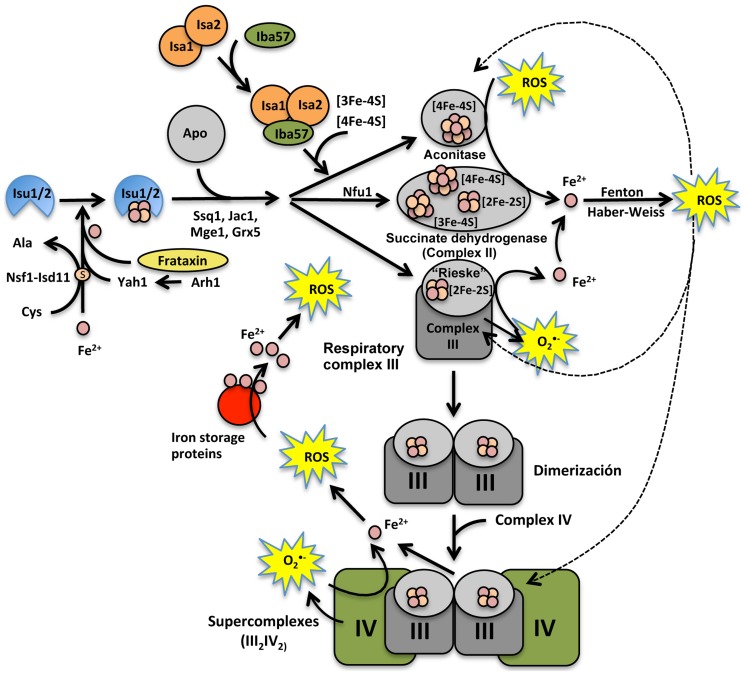
Model proposed for the mechanism of mitochondrial generation of ROS dependent on free Fe^2+^ release from Fe–S-containing proteins in *S. cerevisiae*. The [2Fe–2S] commonly carried by the multi-protein complex (Ssq1-Jac1-Mge1-Grx5) can also be assembled into recipient apoproteins, such as the Rieske protein of cytochrome *bc1* from respiratory complex III. The Isa1 and Iba57 proteins may function as iron reservoirs, from which the metal can subsequently be transferred to [Fe–S] centers or heme prosthetic groups from cytochrome *bc_1_*. When superoxide (O_2_^•−^) is generated by electron leaking in the ETC, and other ROS are produced by oxidative metabolism or by oxidant agents, the [4Fe–4S], [3Fe–4S], or [2Fe–2S] clusters contained in the ETC complexes are disrupted. This event provokes a generalized uncoupling/denaturation of Fe–S proteins, causing a release and thus an increment in the iron labile pool (Fe^2+^), which increases mitochondrial ROS levels via the Fenton and Haber–Weiss reactions. If the ISC assembly system is dysfunctional, supercomplex (III_2_IV_2_) formation is affected, as is [Fe–S] recycling, provoking ETC dysfunction. Hence, the levels of ROS generation increase in an additive manner by a vicious circle of disruption of iron-containing or iron storage proteins, causing an imbalanced ROS content (increment of species such as H_2_O_2_ and superoxide species), that provokes mitochondrial dysfunction and may ultimately lead to apoptotic events.

The findings for Fe^2+^ release in *ssq1*Δ and *isa1*Δ mutants suggest that accumulation of preassembled Fe–S clusters, due to insertion failure caused by disruption of *ISC* or the absence of the target apoproteins (i.e., mitochondrial Fe–S-containing proteins), leads to an increment in the free Fe^2+^ pool, provoking an oxidative stress event (Fig. 3). This may lead to denaturing/dissociation of already assembled Fe–S hemoproteins as an iron source; the prosthetic groups of these proteins could also be one of the iron sources that cause the iron imbalance, leading to a vicious circle of ROS generation (Fig. 10). This is concordant with the impairment of cytochrome *b*_2_, a heme enzyme, observed in all mutants tested. Additionally, it has been reported that in yeast mitochondrial superoxide dismutase (SOD2) can be metallated with iron instead of manganese when iron homeostasis is disrupted, leading to enzyme inactivation. Thus, SOD2 inactivation derived from the intramitochondrial free iron increment may be an additional factor involved in superoxide accumulation, since this mis-metallation is more apparent in Grx5p and Ssq1p mutants, and affects downstream steps in iron-sulfur biogenesis [45], [46].

The fact that excess iron in *ssq1*Δ and *isa1*Δ mutants causes an additive increase in ROS generation (Fig. 3) is in agreement with the increment in O_2_ generation observed in these mutants in the presence of glucose plus 10% ethanol (Fig. 8f). Because the release of Fe^2+^ was enhanced in these mutants, OH^•^ radicals may have also been formed due to the Haber-Weiss cycle. This event would lead to a worsening of the redox state with catastrophic consequences for the cell, due to the high reactivity of OH^•^ with virtually any class of biomolecule. In summary, we suggest that the iron-mediated mechanism of ROS inducer toxicity in *ssq1*Δ and *isa1*Δ mutants may be also be the result of impaired electron transfer at complexes II and IV (Fig. 9 b and 9f), which in turn leads to a reduction in electron transporters in complex III, as reflected by the effect of antimycin A in the Fig. 8f, and further generation of ROS. The increment in ROS may enable the release of more iron from storage systems, and probably from the prosthetic groups of Fe–S or heme proteins, contributing in this way to a feedback mechanism for ROS generation via the increment in the free iron pool (Fig. 10). Moreover, this may be related to an apoptotic phenotype in *ssq1*Δ and *isa1*Δ mutants, observed in the presence of toxic quantities of the ROS inducer, ethanol [20]. These suggestions are supported by the fact that *grx5*Δ, in which lower levels of Fe^2+^ were released in the presence of ethanol, still displayed OCR and partial activity of all ETC complexes, as well as null oxygen release even in the presence of antimycin A (Figs. 8 and 9). The effects of Grx5p deletion suggest that the main function of this protein is the transitory storage of Fe–S clusters during ISC assembly, which is reflected by the lower iron release and resultant lower ROS generation observed in the *grx5*Δ mutant.

Importantly, *isa1*Δ and ssq*1*Δ mutants showed similar phenotypes for ROS susceptibility, ROS generation, Fe^2+^ release, OCR, and ETC complexes activities. We speculate that the free iron released may originate from Fe–S-containing proteins such as ETC complex II, because the *isa1*Δ and *ssq1*Δ mutants were the major producers of ROS, and both completely lack complex II activity (Fig. 9b), suggesting that Fe–S-dependent recipient proteins are involved. These facts also suggest that the Isa1 protein could be involved in *de novo* Fe–S assembly or recycling of Fe-containing proteins from complex II, because Isa1p/Isa2p have also been described as iron reservoirs [12]. However, it is possible that the iron release mainly proceeds from iron storage sources, such as vacuoles or iron-chelation proteins, such as frataxin, which make iron already present within mitochondria available for Fe–S cluster synthesis when iron concentrations are low or the Fe–S content is diminished [16].

With the objective to elucidating this last hypothesis, concerning the roles of Isa1 in Fe–S assembly and iron recycling, three types of possible recipient proteins were evaluated: (1) the [4Fe–4S] cluster from *cis*-aconitase, which has been described as highly sensitive to ROS and which is considered to be an iron donor for the Fenton reaction; (2) the Rieske protein from ETC complex III, which is rich in [2Fe–2S] clusters, and which is recognized as the main source of superoxide generation in mitochondria [47], (3) along with succinate dehydrogenase from complex II, which contains [2Fe–2S], [3Fe–4S], and [4Fe–4S] clusters [48]. As shown in Fig. 6b, *cis*-aconitase was not affected in *atx1*Δ or *mrs4*Δ mutant, but it was affected in *ISC* mutants (*ssq1*Δ and *grx5*Δ), and it was almost totally abolished in the *isa1*Δ strain. Concordant with our results, a reduction in aconitase protein expression and enzymatic activity has been described in *mrs4*Δ iron-transport mutant, along with iron dependence for *de novo* Fe–S cluster formation, dependent on the Mrs3/Mrs4 iron-transporters or frataxin involved in iron homeostasis (its diverse functions include Fe–S clusters synthesis, heme biosynthesis, aconitase repair, respiratory regulation, iron detoxification, iron storage, and oxidative stress protection) [15]–[17]. These findings confirm that in [4Fe–4S] clusters, assembly into target proteins such as *cis*-aconitase is dependent on the Isa1 protein, as described previously [12]. Interestingly, *grx5*Δ mutant showed a diminution in complex II activity, suggesting that assembly of the Fe–S clusters of complex II may also be assisted by the Grx5 protein. Abolishment of succinate dehydrogenase activity (null activity of complex II) in the *isa1*Δ strain strongly suggests its involvement in assembly of Fe–S clusters in the Sdh protein of complex II (Fig. 9b). Interestingly, our Raman spectrophotometry results were in agreement with these observations, indicating that the [2Fe–2S] and [4Fe–4S] cluster content in isolated mitochondria was severely diminished in *isa1*Δ and *ssq1*Δ mutants, compared with the WT. Meanwhile, in the conditional iron-transport mutants *atx1*Δ and *mrs4*Δ, and in *grx5*Δ strain mutants, an increased content of Fe–S clusters was observed (Fig. 6a). Iron-deficiency has been described to provoke a decrease in the iron uptake in *atx1*Δ mutants [31], a condition that causes an increment in iron uptake, as in *mrs4*Δ mutants [17]. This iron-limiting condition induces upregulation of iron-dependent genes controlled by the transcriptional regulator Aft1 [49], and in some genes involved in the ISC system [50]; these observations are concordant with the increased [2Fe–2S] and [4Fe–4S] clusters, corresponding to signals in the Raman spectra of mitochondria from *atx1*Δ, *mrs4*Δ, and *grx5*Δ mutants.

The assembly of ETC supercomplexes formed by cytochrome *bc_1_* complex and cytochrome *c* oxidase (complexes III and IV of the ETC) is dependent on the integration of Rieske protein into the *bc_1_* complex [4], [35], [51]. In this context, we examined formation of ETC supercomplexes III/IV in *S. cerevisiae ISC* mutants using native-blue gels [27]–[30]. We found that supercomplex III_2_/IV_2_ was virtually undetectable in mitochondria isolated from *ssq1*Δ and *isa1*Δ mutants, but supercomplex III_2_/IV_1_ was diminished in both *ISC* mutants, as well as in *grx5*Δ mutants (Fig. 7). In agreement, with these findings, immunoblotting using an anti-Rip1 antibody showed that the ISC system is involved in the assembly of the Rieske subunit of complex III, and that Ssq1p is essential, but Isa1p and Grx5p are not. Moreover, in *grx5*Δ, *atx1*Δ, and *mrs4*Δ mutants, the Rieske protein was up-regulated (Fig. 7c). This result is coincident with the increase in Fe–S cluster signals observed in the Raman spectra (Fig. 6). These findings suggest that the ISC system plays an important role in the assembly of supercomplexes III/IV of the ETC, probably in a Rieske-dependent manner. Alternatively, these findings suggest that these proteins are related to heme biogenesis or assembly in ETC cytochrome-containing proteins such as complex IV or cytochrome *b_5_*, by modulating the bioavailability/recycling of iron. However, further studies are required to corroborate these hypotheses in detail.

We expected that disruption of ISC biogenesis would only impair complexes containing Fe–S clusters. This expectation was concordant with the observed abolishment of complex II activity in *ssq1*Δ and *isa1*Δ mutants, as this complex contains [2Fe–2S], [3Fe–4S], and [4Fe–4S] clusters in the catalytic dimer of the enzyme that play a central role in catalysis, as they receive electrons from FADH_2_ and transfer them to the membrane domain where quinone reduction occurs [24]. Interestingly, the activities of complexes II and III in *grx5*Δ mutants, although decreased, were not totally abolished, as in the other *ISC* mutants; indeed, the activity of complex III with glycerol was similar to that of the WT strain (Fig. 9d). This result seems to contradict the essential role of Grx5p in the activity of complex II, reported by Rodríguez-Manzaneque et al. (2002) [9]. However, this discrepancy may be attributed to the different technique used to measure complex II activity in that study, which involved following the formation of formazan resulting from the reduction of a tetrazolium salt by complex II [52], formazan can also be formed by the reduction of tetrazolium salts by ROS [53]. We avoided this issue by monitoring reduction of DCIP. It has been proposed that Grx5p participates in ISC cluster biogenesis by assisting in the transference of Fe–S clusters from the scaffold to target proteins [54] and/or by repairing mixed disulfides between glutathione and ISC assembly factors [55], in addition, it may constitute transitory Fe–S cluster storage [56], although its exact role remains to be elucidated. Thus, it appears that the function of Grx5p in the assembly of Fe–S clusters from complexes II and III may partially be replaced by other glutaredoxins.

Collectively, these results indicate that the respiratory incompetence of *ssq1*Δ and *isa1*Δ mutants was mainly due to defective oxidation of substrates in complex II and impaired delivery of electrons to complex III, and to oxygen in complex IV.

Although they lack Fe–S clusters, both complex IV and cytochrome *b*_2_ (i.e. both heme-containing proteins) were also affected in all *ISC* mutants, except *grx5*Δ (Fig. 9). This was not at all surprising, since in yeast decreased heme biosynthesis and cytochrome deficiency are general phenotypic features of cells with an impaired mitochondrial ISC system [6]. *ISA1* and *SSQ1* yeast mutants contain decreased amounts of both hemes *c*+*c*_1_ and *b* and residual activity of cytochrome *c* oxidase [57]. Concerning the null complex IV activity of *ssq1* and *isa1*Δ mitochondria, a similar phenotype was reported by Gelling et al. (2008) [10]. The impaired complex IV activity observed in these mutants may also be explained by the inability of this enzyme to form supercomplexes with complex III, as it has been demonstrated that when complex III has an incorrect conformation, the activity of complex IV may be strongly affected [4], [35], [51]. This is in full agreement with the impaired formation of supercomplexes III_2_IV_2_ and III_2_IV_1_ in *ISC* mutants (Fig. 7).

Mitochondrial respiration was absent in *ssq1*Δ and *isa1*Δ mutants in all respiratory states. This effect seems to be mainly attributable to defective electron transfer to O_2_ at complex IV, since this activity was fully inhibited in these cells (Fig. 8). Another factor contributing to the respiratory incompetence of these mutants is their inability to oxidize substrates at complex II, as demonstrated by their observed null succinate-DCIP oxidoreductase and succinate-cytochrome *c* oxidoreductase activities. The impairment in the latter activity was not attributed to defective electron transfer at complex III, since we detected cytochrome *c* reduction in *isa1*Δ in the presence of glycerol at the same level as in WT, but at significantly diminished levels in *ssq1*Δ mutants (Fig. 8). This is concordant with the fact that Isa1p has not been described as participating in the assembly of 2Fe–2S centers, which is the type of ISC present in the Rieske subunit of complex III [5]. However, Raman spectra indicated that a decrease in Fe–S protein content in mitochondria occurs in *ssq1*Δ and *isa1*Δ mutants, also associated with loss or diminution of the Rieske protein in complex III, although the involvement of mitochondrial proteins containing heme groups cannot be neglected. The last observation is in accordance with formation of supercomplexes in the ETC that depend on Fe–S proteins, as seen in the mitochondrial extract from *ssq1*Δ and *isa1*Δ mutants in native gels and western blots, suggesting that, in addition to Fe–S cluster assembly, these proteins could also be involved in heme assembly in mitochondrial respiratory complexes.

Regarding the role of the functionality of ETC in ethanol tolerance, it must be considered that in yeast, mitochondria participate in the maintenance of the redox balance during metabolism of sugars by oxidizing the NADH generated during both glycolysis and ethanol oxidation by the cytosolic and mitochondrial isoforms of alcohol dehydrogenase [58]. This process is very important to avoid deleterious production of mitochondrial ROS, since high NADH/NAD^+^ ratios favor higher rates of ROS production in the ETC [59]. In yeast, the NADH dehydrogenases Nde1, Nde2, and Ndi1 shuttle electrons from NADH to the quinone pool [60], and the ubiquinol generated is oxidized by the quinol-oxidase site of complex III. Although *ssq1*Δ and *isa1*Δ mutants exhibited partial or full complex III activity (Fig. 8), it is possible that the null activities of complexes II and IV indirectly interfere with NADH oxidation and ethanol metabolism. This prevents the re-oxidation of electron acceptors in complex III, which in turn may lead to an increase in the generation of semiquinone radicals, favoring the generation of O_2_^•−^. In accordance with this notion, it has been demonstrated that the inhibition of complex II or complex IV may enhance mitochondrial ROS generation [61], [62]. This point is further supported by the fact that the addition of antimycin A leads to ROS generation (Fig. 8c and 8f). This also indicates that ubiquinol is being oxidized at the quinol oxidase (Qo) site of complex III for bifurcated reduction of cytochrome *b* and posterior O_2_^•−^ formation by inhibition of re-oxidation of cytochrome *b*_562_ in the quinone reductase site (Q_i_) induced by antimycin A [24]. Otherwise, the generation of O_2_ by antimycin A would not be possible. Furthermore, the exacerbation of O_2_ release by antimycin A with ethanol treatment (Fig. 8) is suggestive of further impairment of the Q cycle in complex III, corroborating the hypothesis that the toxicity of ROS inducers is mediated by altered electron transfer in the ETC, leading to enhanced ROS generation. It must be stressed again that O_2_ generation is an indicator of ROS production because O_2_ is a product of the degradation of O_2_^•−^ and H_2_O_2_, catalyzed by superoxide dismutase and catalase, respectively. Importantly, we also found that these mutants have increased catalase activity [20], which may be an adaptive response to enhanced ROS generation due to impaired ETC function.

In summary, exacerbation of ROS generation in *S. cerevisiae* caused by treatment with stressors such as ethanol, H_2_O_2_, and menadione, occurs via Fe^2+^ release, which is favored by an iron-dependent ROS generation cycle. Microscopic analysis of the WT strain showed that free Fe^2+^ release and ROS co-localized mainly in mitochondria, and were exacerbated by ethanol treatment (a ROS inducer), whereas in *ssq1*Δ mutants, both free Fe^2+^ and ROS were observed in all cells. This pattern was also observed in *atx1*Δ and *mrs4*Δ mutants, which are hyper-iron accumulators in an iron-rich media. Interestingly, a phenotype of bloated vacuole structures was observed in *ISC* mutants, as in iron-accumulator mutants (*atx1*Δ and *mrs4*Δ), suggesting dysfunctional iron homeostasis associated with mitochondria and vacuole organelles. Raman spectroscopy and supercomplex formation of mitochondria isolated from *ISC* mutants indicated that disruption of Ssq1 and Isa1 proteins provoked a decrease in [2Fe–2S] and [4Fe–4S] cluster content that was reflected in loss of the Rieske protein from complex III and disrupt supercomplex formation between complexes III and IV, leading to dysfunction of the ETC and probably to mitochondrial apoptotic events.

Our findings indicate that free Fe^2+^ release and ROS generation are interdependent and are associated with mitochondrial iron homeostasis, via Fe–S-containing proteins, and with storage/detoxification systems, such as frataxin, which are important iron sources. The bloated vacuoles observed in *ISC* mutants following treatment with ROS inducers, as well as in iron-accumulator mutants (*atx1*Δ and *mrs4*Δ) suggest that an iron imbalance occurred was important in the loss of iron homeostasis, that in turn contributed to ROS generation, and to impaired Fe–S cluster biogenesis of proteins from the ETC. The oxidative stress generated and the effects on the Fe–S-containing proteins led to mitochondrial dysfunction.

## Supporting Information

Figure S1 **Studies of growth in plates of the** ***S. cerevisiae ISC*** **mutants.** A–D) Dilutions of yeast suspensions were cultured on YPD agar plates with or without ROS-inducers at the indicated concentrations at 30°C for 48 h. Yeast cultures grown on YPD medium plates with: A) different concentrations of the iron chelator 1,10-phenanthroline (10 µM and 20 µM). B) low-iron content using the iron chelator 1,10-phenanthroline (20 µM) and different concentrations of ferrous iron (5–20 µM). C) ROS-inducers at indicated concentrations of H_2_O_2_ (4 mM), menadione (80 µM), and ethanol (8%). D) low-iron content using 1,10-phenanthroline (20 µM) plus ferrous iron (20 µM) with the concentrations indicated of ROS-inducers H_2_O_2_ (4 mM), menadione (80 µM), and ethanol (8%). E) low-iron content using 1,10-phenanthroline (20 µM) plus ferrous iron (500 µM) with the concentrations indicated of ROS-inducers H_2_O_2_ (4 mM), menadione (80 µM), and ethanol (8%).
(TIF)
