# Supplementary figures and images for: Malfunctioning of the Iron–Sulfur Cluster Assembly Machinery in Saccharomyces cerevisiae Produces Oxidative Stress via an Iron-Dependent Mechanism, Causing Dysfunction in Respiratory Complexes
Source: PLoS One. 2014 Oct 30;9(10):e111585. doi: 10.1371/journal.pone.0111585 (PMC4214746; doi:10.1371/journal.pone.0111585)

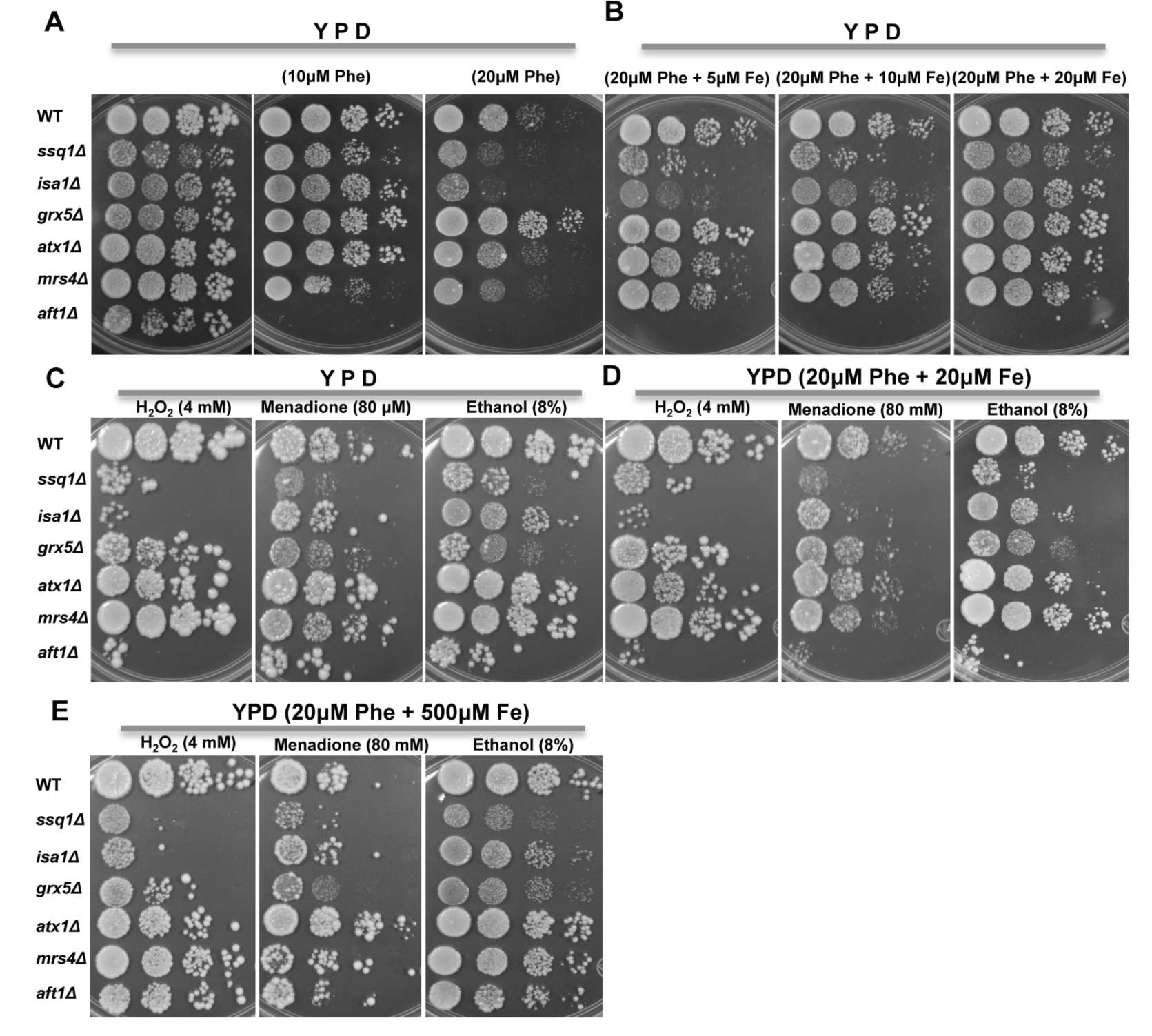

Supplement: Figure S1 — Studies of growth in plates of the S. cerevisiae ISC mutants. A–D) Dilutions of yeast suspensions were cultured on YPD agar plates with or without ROS-inducers at the indicated concentrations at 30°C for 48 h. Yeast cultures grown on YPD medium plates with: A) different concentrations of the iron chelator 1,10-phenanthroline (10 µM and 20 µM). B) low-iron content using the iron chelator 1,10-phenanthroline (20 µM) and different concentrations of ferrous iron (5–20 µM). C) ROS-inducers at indicated concentrations of H2O2 (4 mM), menadione (80 µM), and ethanol (8%). D) low-iron content using 1,10-phenanthroline (20 µM) plus ferrous iron (20 µM) with the concentrations indicated of ROS-inducers H2O2 (4 mM), menadione (80 µM), and ethanol (8%). E) low-iron content using 1,10-phenanthroline (20 µM) plus ferrous iron (500 µM) with the concentrations indicated of ROS-inducers H2O2 (4 mM), menadione (80 µM), and ethanol (8%). (TIF) [file pone.0111585.s001.tif]
